# Retention in trials: a qualitative evidence synthesis of studies reporting participant reasons for trial non-completion

**DOI:** 10.1136/bmjopen-2025-111824

**Published:** 2026-04-20

**Authors:** Ellen Murphy, Katie Gillies, Zoe Skea, Linda Biesty, Andrew Hunter, Nurulamin M Noor, Sharon McCann

**Affiliations:** 1 Trials Research and Methodologies Unit (TRAMS), Health Research Board Clinical Research Facility, University College Cork, Cork, Ireland; 2 Trials Methodology Research Network (HRB-TMRN), Health Research Board, Dublin, Leinster, Ireland; 3 Aberdeen Centre for Evaluation, University of Aberdeen, Aberdeen, UK; 4 School of Nursing & Midwifery, University of Galway, Galway, Ireland; 5 Evidence Synthesis Ireland, University of Galway, Galway, Ireland; 6 Department of Medicine, University of Cambridge, Cambridge, UK

**Keywords:** QUALITATIVE RESEARCH, Randomized Controlled Trial, Review

## Abstract

**Abstract:**

**Objectives:**

Poor participant retention in randomised clinical trials, resulting in missing outcome data, can impact the validity, reliability and generalisability of results. While participants’ views on general non-retention issues have been reported elsewhere, a qualitative evidence synthesis specifically focusing on trial processes (ie, outcome data collection) impacting retention has not been undertaken to date. This is an important research question to inform targeted interventions to support retention. This review aims to address this by systematically searching and synthesising the evidence on participant reasons for trial non-completion, linked to outcome data collection.

**Design:**

We conducted a qualitative evidence synthesis of qualitative studies and mixed methods studies with a qualitative component, in Embase, Ovid MEDLINE, PsycINFO, Cochrane Central Register of Controlled Trials (CENTRAL), Social Science Citation Index, Cumulative Index of Nursing & Allied Health Literature and Applied Social Sciences Index and Abstracts, up to February 2025. We used Thomas and Harden’s thematic synthesis approach. The Grading of Recommendations Assessment, Development and Evaluation-Confidence in the Evidence from Reviews of Qualitative framework was used to assess confidence in the review findings.

**Participants:**

We identified 11 studies reporting qualitative data from 14 separate trials, with findings from 105 trial non-retainers. The studies were undertaken between 2007 and 2025.

**Results:**

There were three types of participant non-retention behaviours reported across the studies, where participants either: (1) missed at least one clinic visit; (2) did not complete a postal questionnaire or (3) did not complete online data collection. We developed four analytical themes outlining participant-reported influences on trial non-retention, specifically related to trial processes (ie, data collection for outcome measures): fluctuating health, balancing trial burdens, navigating life as a trial participant and managing expectations of participation.

**Conclusions:**

This review generates important insights into participants’ reasons for trial non-completion linked to outcome data collection. The review highlights the need for further research into supporting trial recruitment discussions that provide clear, realistic expectations for potential trial participants, as well as strategies that recognise, and where possible, address some of the influences on participants to improve outcome data completeness and ultimately improve trial retention.

**STRENGTHS AND LIMITATIONS OF THIS STUDY**

- This synthesis was conducted using established, standardised and reproducible qualitative evidence synthesis methods.
- All the included studies were recently published (2007–2025) and therefore findings capture and reflect the most recently published literature on the topic.
- We used the Grading of Recommendations Assessment, Development and Evaluation-Confidence in the Evidence from Reviews of Qualitative approach to provide a transparent assessment of our confidence in the review findings.
- The synthesis included 11 eligible studies, from 14 separate trials, predominantly in high-income settings which may limit the transferability of findings.

## Background

 Retention (ie, ensuring that trial participants remain in a trial to provide primary outcome data) in randomised controlled trials (RCTs), herein referred to as ‘trials’, is an ongoing challenge for trial teams. Due to the implications of poor retention, it has been identified as an area of priority for methodological research by a consensus priority setting partnership group of those working in the field of trials.^1^ Trials commonly experience problems with missing data often referred to as a ‘loss to follow-up’, ‘attrition’ or ‘drop out’.^2 3^ This means that trials often experience non-completion of outcome data collection (ie, fail to obtain complete primary outcome data) from participants, a problem that has important potential implications for utility of results from these trials.^4^ Poor retention rates cause issues of bias, as well as concerns regarding the validity, reliability and confidence in the results of a trial.^4^,^7^ There are many statistical methods that can be routinely used to impute data from participants who do not remain in trials, but even use of these methods can lead to inaccurate results.^4 6 8^ Substantial loss to follow-up can sometimes lead to either overestimations and underestimations of the effects of an intervention.^4^ Inadequate planning to address these retention issues can make recruitment even more difficult and can contribute to the growing problem of research waste.^9^

To address the issue of poor retention, it is crucial to understand why participants discontinue trial involvement either deliberately (eg, intentionally not completing some, or all, follow-up requirements or active withdrawal, eg, calling to request no further information is sent) or passively (eg, forgetting to attend a clinic visit or return a follow-up questionnaire). Understanding participants’ reasons for trial non-retention is important for identifying potentially modifiable aspects of trials to optimise ongoing participation.

A meta-ethnography by Skea *et al*,^10^ was the first to synthesise qualitative evidence on participant-reported factors influencing non-retention in trials, focusing on intervention adherence in addition to trial processes. Participant reasons for trial non-completion included concerns about trial medication such as its unlicensed status, unpleasant taste, fears about excessive medication taking and unease about trial medication interference with other usual medication. Participants’ perceptions of their health also influenced trial retention. Some withdrew when their health improved, feeling ongoing participation was unnecessary, while others withdrew when their health declined, as they felt there was no longer a reason to remain in the trial, or were too ill to continue in the trial. Trial non-completion also occurred if the trial did not meet participants’ care expectations or when activities were incompatible with their capabilities, such as limited reading and writing skills. Participants who struggled to accept their illness diagnosis could find it difficult to relate or find value in the trial intervention, and fitting trial participation around work and family commitments was also challenging.^10^

### Rationale for this qualitative evidence synthesis

The Qualitative Evidence Synthesis (QES) reported in this paper advances the discussions generated by the Skea *et al*^10^ meta-ethnography, with a focus on trial non-completion linked to trial processes (ie, data collection for outcome measures). We are also aware of key studies that have been published on participant reasons for trial non-completion since the Skea *et al*^10^ meta-ethnography was conducted.^11 12^

While it is important to understand reasons for non-completion of trials linked to trial interventions, our review specifically focuses on trial non-completion arising from outcome data collection and its impact on retention. Understanding these specific behaviours related to data collection could help target specific strategies to improve retention.

Therefore, the aim of the QES was to systematically search and synthesise the qualitative evidence on participant reasons for trial non-completion, linked to outcome data collection.

## Methods

This is a QES review and is reported in accordance with the ENTREQ statement^13^ (completed checklist online supplemental file 1) and the Preferred Reporting Items for Systematic Reviews and Meta-Analyses (PRISMA) guidelines.^14^ The review methods were guided by the QES chapter in the Cochrane Handbook,^15^ as well as guidance on updating meta-ethnographic syntheses.^16^ Following the Research question, Epistemology, Time/Timeframe, Resources, Expertise, Audience and Purpose, Type of data (RETREAT) framework,^17^ for selecting review methodologies, Thomas and Harden’s thematic synthesis approach was used to analyse and synthesise the data.^18^ The confidence in the findings of this review was assessed using the Grading of Recommendations Assessment, Development and Evaluation-Confidence in the Evidence from Reviews of Qualitative Research (GRADE-CERQual)^19^ (online supplemental file 2).

### Eligibility criteria

We used the SPIDER (Sample, Phenomenon of Interest, Study Design, Evaluation and Research Type) framework^20^ to help identify the main concepts of our review and guide our eligibility criteria. Table 1 outlines each of the SPIDER domains for this QES. The full list of terms used to search the various databases is provided in online supplemental file 3.

**Table 1:** SPIDER framework

Sample	All studies exploring trial participants’ accounts of their reasons for not completing randomised clinical trials/randomised controlled trials in a clinical setting.
Phenomenon of Interest	Trial participants’ reasons for not completing some or all the data collection processes for trial outcome measures. Non-retention might cover activities such as non-attendance at clinic visits, or non-response to some or all follow-up questionnaires for outcome assessment, etc. Reasons for non-retention had to be linked specifically to trial processes, that is, data collection for outcome measures.
Design	Primary qualitative studies (eg, ethnography, grounded theory studies) that use recognised methods of qualitative data collection (eg, focus groups, interviews). Mixed methods studies, if data were collected and analysed using qualitative methods, and qualitative data can be extracted.
Evaluation	Reasons, barriers, experiences, views
Research type	Qualitative or mixed methods. Published literature, grey literature. No restrictions were placed on age, social status, ethnic background, country of recruitment. Studies had to be published in the English language.

SPIDER, Sample, Phenomenon of Interest, Study Design, Evaluation and Research Type.

### Information sources

We conducted an expansive search of the literature^21^ for qualitative studies and mixed methods studies with a qualitative component, using the following electronic databases:

Embase, Ovid MEDLINE, PsycINFO, the Cochrane Central Register of Controlled Trials (CENTRAL), the Social Science Citation Index, Cumulative Index of Nursing & Allied Health Literature and Applied Social Sciences Index and Abstracts. In addition, we manually searched a reference list of an unpublished PhD thesis on participant retention in trials.^22^

### Search strategy

The search strategy combined the five concepts of the SPIDER framework^20^ and was developed by KG and LB and peer-reviewed by the team, including a health information specialist. The search strategy is in online supplemental file 3. No language, geographic or publication date restrictions were applied to the search.

The final list of citations for the identified studies was exported to EndNote, and duplicates were removed. References were then imported to Excel to facilitate screening. The initial search was conducted in October 2021, followed by an update in May 2023. To ensure findings were contemporaneous, a further update was conducted in February 2025 to identify any new papers which were synthesised in findings as relevant. All three searches were combined and presented in online supplemental file 3.

### Study selection

**Figure 1:**
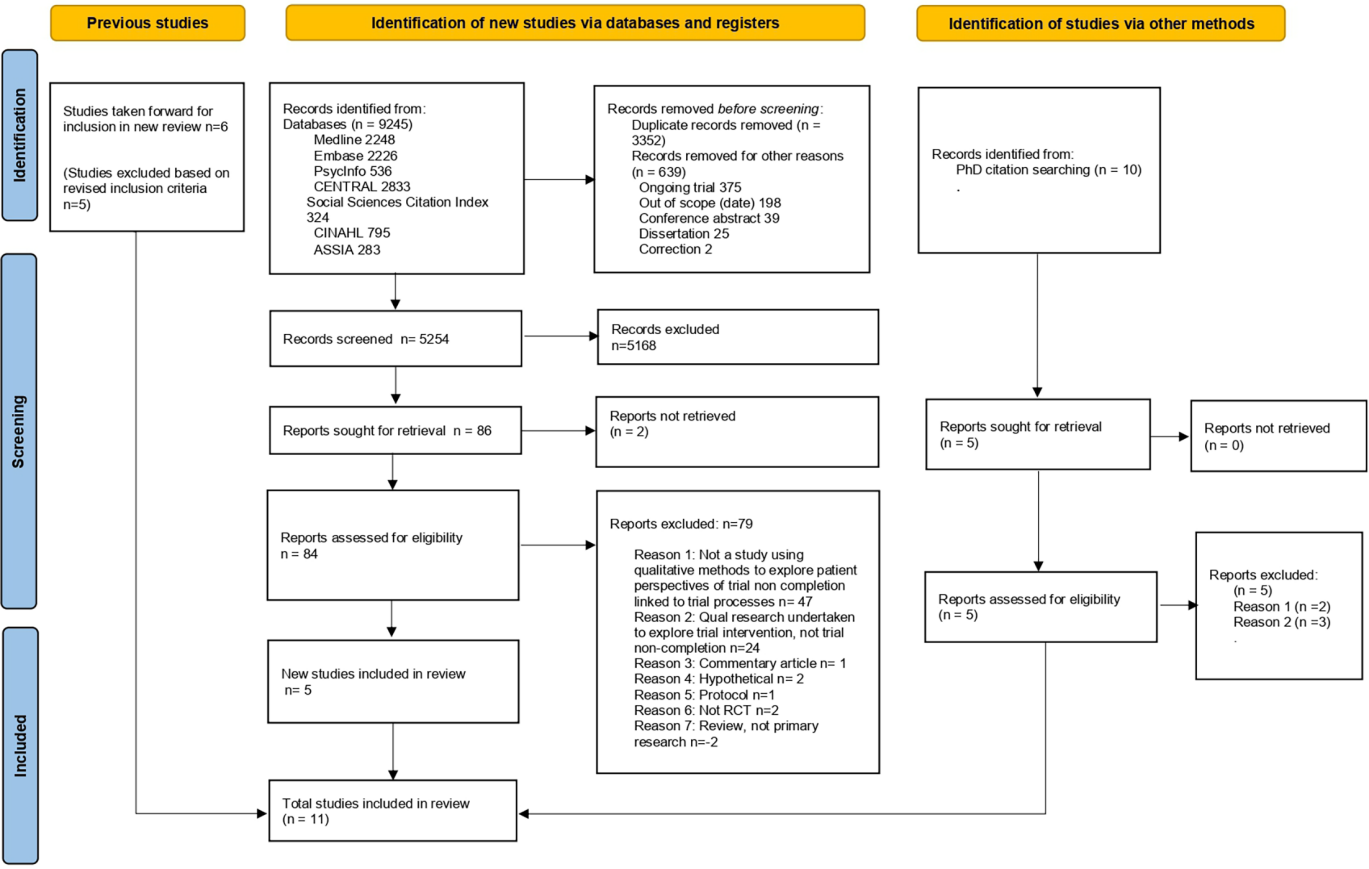
PRISMA flow diagram. PRISMA, Preferred Reporting Items for Systematic Reviews and Meta-Analyses; RCT, randomised controlled trial.

All titles and abstracts were screened to determine if the predefined set of inclusion criteria outlined by SPIDER^20^ were met. All review authors independently single screened a batch of titles and abstracts, and a random 10% check of all screened titles and abstracts was completed by EM. KG resolved any disagreements. Full texts of papers potentially meeting the inclusion criteria were sourced, and full text screening was conducted independently by SM and EM. Another review author (KG) resolved any disagreements. The number of studies at each stage of the screening process and reasons for exclusions are presented using a PRISMA flow diagram in figure 1.

As the aim of this QES was to identify participants’ reasons for non-retention linked to trial processes ie, data collection for outcome measures, the 11 studies included in the Skea *et al*^9^ meta-ethnography were re-assessed against our eligibility criteria. As such, studies that did not report on reasons for trial non-completion linked to trial processes were excluded (n=5). Results of these are outlined in the PRISMA flow diagram (figure 1).

### Data extraction

Two review authors (SM and EM) extracted data from all included studies using a predesigned data extraction form, adapted and expanded from the Skea *et al* QES,^10^ to focus on participants’ reasons for trial non-completion specifically related to outcome data collection. Information extracted included characteristics of the study (design, data collection and analysis methods) (online supplemental file 4). All qualitative findings from the primary studies relevant to the research question were extracted. During data extraction, EM and SM had to carefully re-read the included papers multiple times to ensure that only information about non-retainers for reasons linked to trial processes was extracted, consulting with KG as needed, to determine whether reasons and participant characteristics were clearly attributable to those who were non-retainers linked to trial processes.

The characteristics of included studies table is presented in online supplemental file 5. We extracted data relating to the aim of this QES, such as themes and verbatim text extracts included in the individual studies relating to participant-reported reasons for trial non-retention. Data were extracted for both participant quotes (ie, first order constructs) and the study authors' interpretation (ie, second order constructs), statements, assumptions and ideas, to help ensure that the review findings were fully grounded in the original experiences of trial participants. A table showing all the data extracted as first and second order constructs is presented in online supplemental file 6.

### Assessing the methodological limitations of included studies

The methodological limitations of the included studies were appraised using the Critical Appraisal Skills Programme (CASP) tool,^23^ which offers a suitable framework to assess the methodological limitation of primary qualitative studies. Studies were appraised independently by three reviewers. No studies were excluded on the basis of quality assessment, but the quality assessment informed the subsequent confidence of findings assessment.^19^ The CASP assessment for included studies is outlined in online supplemental file 7.

### Data synthesis

Guided by the RETREAT framework,^17^ a thematic synthesis method, as outlined by Thomas and Harden,^18^ was used to analyse and synthesise extracted data. This method involved line-by-line coding, creating descriptive themes and generating analytical themes.^18^

Line by line coding of all included studies was carried out independently by two reviewers (SM and EM). Initial themes were discussed with other team members and concepts were discussed and agreed. NVivo software V.14^24^ was used to support coding, check for similarities, differences and consistency of interpretation across the coded text. This iterative process facilitated the next stage of ‘translation’. Codes were grouped into overarching themes with subthemes, to support analytical development with team members. A draft summary of findings across the included studies was undertaken by SM and EM and shared with all review authors to facilitate collective interpretation. Up to this stage, the themes reflected descriptive accounts of the original findings. Following team discussions, SM and EM then developed more analytical themes to move beyond the original data, consistent with the approach outlined by Thomas and Harden.^18^ Through ongoing team discussions, constant comparison of NVivo codes and refinement of conceptual groupings, broader analytical themes were generated to encapsulate all the descriptive themes.

### Assessment of confidence in the findings of this review

The GRADE-CERQual^19^ was used to assess the confidence that the review team had in each finding. The GRADE-CERQual approach is based on four components: (1) methodological limitations of included studies; (2) coherence of review findings; (3) adequacy of data contributing to review findings and (4) relevance of included studies to the research question.^19^ A summary of findings was derived from the thematic synthesis findings, summarising the core idea of each finding, which was subsequently used to inform an overall GRADE-CERQual assessment of confidence based on those findings. The summary is shown in table 2, and a detailed evidence profile is presented in online supplemental file 2. The overall assessment (high, moderate, low or very low confidence) was based on discussion and agreement among EM, SM and LB. Judgements were based on an initial assumption of ‘high confidence’ and then downgraded if concerns arose regarding any of the GRADE-CERQual components and agreed on with the broader team.

**Table 2:** Summary of GRADE-CERQual assessment

Summary of review finding	Studies contributing to review finding	GRADE-CERQual assessment of confidence in the evidence	Explanation of GRADE-CERQual assessment
Theme 1: Fluctuating health	Henshall, Keghia, Wells, Nakash, Lawrie, Nicholas, Postel, Sanders		
1.1 Coming to terms with a diagnosis Some participants struggled with the timing of recent health diagnosis/condition concurrent to trial participation. Feelings of overwhelm, being confronted and the emotional impact of dealing with a diagnosis/condition were reported as making it especially difficult for some people to engage with trial information. Disruption to routine care and health service provision was also reported as impacting on non-retention	Henshall, Wells, Nicholas, Sanders	Moderate confidence	Minor concerns about methodological limitations. Moderate concerns about adequacy and relevance.
1.2 Changeability to health Participants reported aspects of changes to their health (positive and negative) during trial engagement. Changeability was reported as influencing trial non-retention related to follow-up outcome data collection, for example, not seeing relevancy of completing a follow-up questionnaire if feeling their health had improved.	Keghia, Nakash, Lawrie, Nicholas, Postel	Moderate confidence	Minor concerns about methodological limitations and relevancy. Moderate concerns regarding adequacy.
Theme 2: Balancing trial burdens	Henshall, Magazi, Newlands, Wells, Nakash, Lawrie, Draper		
2.1 Personal costs associated with trial participation Participants reported the burdensome nature of trial participation in terms of personal opportunity costs directly linked to participating in a trial. Issues such as childcare, time off work and travel costs were specifically reported as influencing trial non-retention. These issues were exacerbated for participants in low middle income countries.	Henshall, Magazi, Newlands, Wells, Nakash, Draper	Moderate confidence	Minor concerns about methodological limitations and coherence. Moderate concerns about adequacy.
2.2 Type and timing of data collection Participants reported barriers to data collection specifically related to type and timing of data requirements, which impacted trial retention. The majority of participants reported on data collection linked to follow-up questionnaires.	Henshall, Newlands, Nakash, Lawrie	Moderate confidence	Minor concerns about methodological limitations. Moderate concerns about adequacy and relevance.
2.3 Strategies to support continued participation Participants reported their personal attempts to support completion of outcome data assessments as well as attempts by trial teams. Although strategies were reported as facilitating some participants at various stages, ultimately, attempts to support trial retention were not successful.	Lawrie, Magazi, Newlands, Nakash, Wells, Draper	Moderate confidence	Minor concerns about methodological limitations and relevance. Moderate concerns about adequacy.
Theme 3: Navigating life as a trial participant	Magazi, Newlands, Wells, Nakash, Nicholas, Postel, Draper		
3.1 Life gets in the way Participants shifting life priorities and commitments outside of the trial, for example, moving house, college and pregnancy, interfered with ongoing trial participation and completion of outcome assessments.	Nicholas, Nakash, Magazi, Postel, Wells, Newlands, Draper	Moderate confidence	Minor concerns about methodological limitations. Moderate concerns about adequacy.
3.2 Perceptions of self Participants’ own perceptions of themselves, and their capabilities can potentially undermine the completion of data collection activities. Personal feelings of anxiety and fear over aspects of completing data collection were also cited as a barrier to follow-up completion.	Nakash, Nicholas, Newlands,	Moderate confidence	Minor concerns about methodological limitations. Moderate concerns about adequacy and relevance.
3.3 Cultural community context Cultural context and the impact of community and family influenced non-retention in some trials in low-income settings.	Magazi, Wells, Draper	Moderate confidence	Moderate concerns regarding adequacy of data. Moderate concerns about relevance.
Theme 4: Managing expectations of participation	Magazi, Newlands, Wells, Nakash, Lawrie, Draper		
4.1 Expectations of care and relational support offered by trial participation Not meeting participants’ expectations regarding care and support from trial staff, and participants feeling their participation in the trial is not valued or acknowledged, was reported as impacting on trial non-retention.	Magazi, Newlands, Wells, Lawrie, Draper	Moderate confidence	Minor concerns about methodological limitations and relevancy. Moderate concerns with adequacy.
4.2 Understanding what is expected as a trial participant Participants often were unclear about follow-up requirements, the implications of not completing follow-up, and questioning their personal contribution and value to the study. Some participants reported confusion about being labelled as a ‘drop-out’.	Wells, Nakash, Lawrie, Newlands	Moderate confidence	Minor concerns regarding methodological limitations, coherence and relevance. Moderate concerns with adequacy.

GRADE-CERQual, Grading of Recommendations Assessment, Development and Evaluation-Confidence in the Evidence from Reviews of Qualitative.

## Results

### Search and study selection

The three electronic database searches (October 2021, May 2023, February 2025) yielded 9245 records. Duplicates were removed using Endnote, and 5254 remaining papers underwent title and abstract screening against the inclusion criteria. 5168 were excluded at this stage. The remaining 86 were sought for retrieval and full-text screening. In addition, we identified 10 papers from a PhD thesis, which underwent title and abstract screening. Five were excluded, and the remaining five were sought for retrieval and full-text screening. In total, 11 papers (reporting across 14 separate trials) were included in the review (see figure 1 for details).

### Description of included studies

A summary of the characteristics of the studies included in the QES is provided in online supplemental file 5. This summary reflects how the study authors described participants and the trials in their studies.

In total, 11 studies (across 14 separate trials) met our inclusion criteria. There were three types of participant non-retention behaviours reported across the studies, where participants either: (1) missed at least one clinic visit^1225^,^29^; (2) did not complete a postal questionnaire^11 12 30^; or (3) did not complete online data collection.^31^,^33^ The included studies were conducted in 5 countries (the UK,^11 12 26 29 30 33^ Australia,^31^ the Netherlands,^32^ the USA^27^ and South Africa,^25 28^ across 14 separate trials). The number of non-retained participants specifically linked to issues of data collection across the studies varied. In two papers, the number of participants not retained due to trial-related processes was explicit^11 12^; however, in the remaining studies, closer examination of the reported data had to be conducted to identify participants who were non-retainers (for reasons linked to data collection) from participants who were non-retainers for reasons linked to the intervention. The total number of non-retainers identified was 105.

The characteristics of all participants were provided in the individual studies; however, the specific characteristics (age, gender, ethnicity, etc) of those not retained, for reasons linked to trial processes, were not always clear in studies reporting on both retained and non-retained participants. The number of participants identified within each primary study as being non-retained for reasons linked to trial processes, primary study and participant characteristics is outlined in online supplemental file 5.

All but one study^12^ included participants from a single trial. The included studies were set in trials from a range of different clinical contexts—acute injury—severe ankle sprains,^30^ mental health,^27 31^ alcohol addiction,^32^ type I diabetes,^29^ diabetes, chronic obstructive pulmonary disease, heart failure,^33^ HIV,^25^ uncomplicated symptomatic gallstones disease,^11 12^ insomnia disorder, urodynamic stress incontinence, ureteric stones, dental health,^12^ Parkinson’s disease^26^ and maternal health.^28^

The types of interventions evaluated within the host trials varied. These interventions included drug,^25 26^ surgical,^11 12^ behavioural,^12 29 31 32^ device,^12 30 33^ mixed interventions^27^ and a complex intervention.^12 28^ Details of the individual interventions are outlined in online supplemental file 5.

Various qualitative methods were used to collect data across the studies; interviews (face-to-face interviews,^25 26 29 30^ in-depth telephone interviews,^27^ semistructured telephone interviews,^1112 29^,^31 33^ serial ethnographic interviews,^25^ focus group discussions^25 26 28^ and questionnaires with open-ended questions).^26 32^

### Assessment of the methodological limitations of included studies

The methodological limitations were assessed. We only had minor concerns about the methodological quality of the eleven included studies, primarily due to limited reporting of participant data, reflexivity and ethics. (Full details are available in online supplemental file 7).

### Confidence in the review findings

We developed four main themes outlining factors influencing participant

reasons for trial non-completion. 10 key findings are presented within these themes.

We conducted a GRADE-CERQual assessment on the 10 findings. The summary is shown in table 2 and a detailed evidence profile, including assessment rationale, is presented in online supplemental file 2. Our confidence in the findings was downgraded primarily because of concerns about adequacy and/or relevancy issues, such as findings based on a small number of studies, from a limited geographical setting, or due to the thinness of data reported. There were some minor methodological concerns, related mainly to a lack of evidence about research reflexivity and the documentation of ethical considerations.

### Reflexive note

The review team included researchers with a range of experiences in trial design, conduct and retention research, which will have shaped the review processes. To mitigate interpretive bias, we held ongoing team discussions, reflected on how our backgrounds might shape the synthesis, and considered alternative explanations when comparing concepts across studies. Throughout the review, we also documented the synthesis stages, team discussions and rationale for analytic and methodological decisions to enhance transparency and ensure that the resulting interpretations remained grounded in the data. Core review activities were undertaken by four authors (EM, SM, KG and LB) with all authors contributing to ongoing interpretation and synthesis through regular team meetings and shared online resources.

### Synthesis and findings

**Figure 2:**
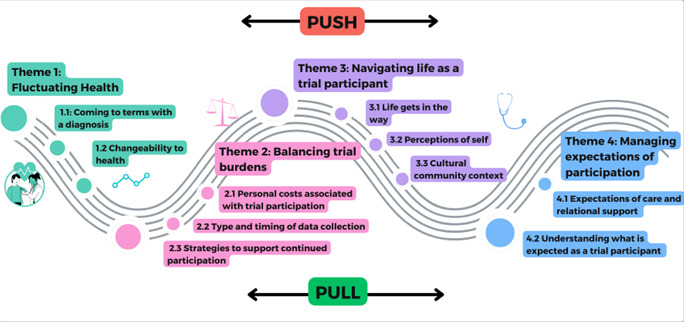
‘Push and pull’ factors that impact on participant trial non-retention.

We identified four analytical themes relating to the review aim of understanding participant-reported influences on trial non-retention, specifically related to trial processes (ie, data collection for outcome measures). The four themes are: fluctuating health, balancing trial burdens, navigating life as a trial participant and managing expectations of participation (table 3). These were linked by an overarching theme reflecting the fluidity of ‘push and pull’ influences on participants’ reported experiences and influences on trial non-retention. We have created a visual of these ‘push and pull’ factors in figure 2. Each of the four analytical themes is described below using data from the included studies to illustrate key findings. For each finding, we report our confidence by presenting our GRADE-CERQual assessment. The detailed evidence profile, including the studies contributing to each finding and our assessment rationale, is provided in online supplemental file 2.

**Table 3:** Overview of analytical themes

Overarching theme	Analytical theme	Key findings
Push and pull influences	1. Fluctuating health	1.1 Coming to terms with a diagnosis 1.2 Changeability to health
	2. Balancing trial burdens	2.1 Personal costs associated with trial participation 2.2 Type and timing of data collection 2.3 Strategies to support continued participation
	3. Navigating life as a trial participant	3.1 Life gets in the way 3.2 Perceptions of self 3.3 Cultural community context
	4. Managing expectations of participation	4.1 Expectations of care and relational support offered by trial participation 4.2 Understanding what is expected as a trial participant

Where participant characteristics are provided for participants in the primary study, this is shown after each participant quote presented in this paper (to protect participant anonymity, we reported age using broad categories eg, ‘mid-30s’ rather than exact ages). We included an identifier to indicate whether the participant has missed a clinic visit, or non-completion of postal questionnaires or online data collection. Additionally, for all quotes presented, we indicate whether the quote was a primary participant quote (first order constructs) or the study author’s interpretation reported in the primary study (second order constructs).

### Theme 1: fluctuating health

This theme highlights the issues related to participants’ health status concurrent to trial participation and how changes over time impacted trial non-retention.^1126 27 29^,^33^ Findings encompassed two subthemes relating to: (1) participants’ experiences of coming to terms with a diagnosis or illness and (2) changeability to their health (physical and/or mental) during a trial and how fluctuations in these influenced trial non-retention.

#### Subtheme 1.1: coming to terms with a health diagnosis

Narratives about struggling to come to terms with a health diagnosis and its emotional impact were reported as impacting on trial retention.^27 29 31 33^ For example, participants described feeling ‘confronted’ by a diagnosis or struggling to accept their illness, which made trial participation more difficult:

Nicholas *et al*^31^ [page 9] (first order construct): “I found it quite confronting and reading the information made me feel uncomfortable, thinking that these issues related to me—I preferred the ostrich approach.” (Male, 40-49 years, bipolar disorder, BEP group) (online data collection).

Part of coming to terms with a health diagnosis alongside participating in a trial could mean potential changes to a participants’ ‘usual care’ prior to trial participation, which could be stressful and disruptive^33^:

Sanders et al^33^ [page 9] (first order construct/second order construct): “ Another man (ID 92) described the good care he received prior to joining trial, but how he was subsequently discharged from the specialist professionals who had been involved in his care. Whilst he was entered into the trial for his diabetes, he described his main problems as ‘complex problems with my heart and breathing’ (ID 92) and that the faulty recordings and changes in service provision were causing him great stress.” (online data collection)*.*

#### Subtheme 1.2: changeability to health

Fluctuations in people’s health status reflected a fluidity that impacted trial retention in various ways across different timepoints.^1126 30^,^32^ While some studies reported participant perceptions of improvement in their health status, others reported no change or, in some cases, a deterioration in their health. Such changeability could interfere with people’s ability or willingness to continue in a trial and completion of some follow-up outcome assessments.

For example, being in an acute stage of an illness during a trial or where symptoms had worsened was cited by some participants as having an impact on their well-being and abilities to perform basic day-to-day tasks, making trial engagement unsustainable:

Nicholas et al^31^ [page 9] (first order construct): “The biggest problem I have with my bipolar disorder is consistency ; when I’m down I can’t even brush my teeth or get up in the morning.” [Female, 18-29years*,* BEP group] (online data collection)*.*

Similarly, when participants reported their health had improved or that they had partly recovered during trial participation, this could also interfere with completing follow-up assessments,^1130^,^32^ as participants described no longer needing or having anything to ‘offer’ the trial:

Nicholas *et al*^31^ [page 9] (first order construct): “Things really improved for me…I just felt really good and didn’t really feel like I had that much to offer in regard to finding out more about it” [Female, 30-39yrs, control group] (online data collection).

Nakash *et al*^30^ [page 230] (second order construct) “Almost half of the participants who did not respond to follow-up considered themselves to have made a full recovery by the second follow-up point.” (postal questionnaires).

### Theme 2: balancing trial burdens

This theme describes participants’ experiences of balancing trial participation with personal and/or work costs (directly related to trial participation), particularly if required to attend trial clinic appointments for data collection purposes.^1112 25 27^,^30^ Findings comprise three sub-themes related to: (1) personal costs associated with trial participation; (2) type and timing of data collection; (3) strategies used by participants to support data collection completion (including the extent to which trialists were perceived to support (or not) participant retention), and how these impacted trial non-retention.

#### Subtheme 2.1: personal costs directly associated with trial participation

Participation could be potentially burdensome for some participants in terms of personal costs directly associated with trial involvement. Specifically, personal costs attributed to attendance at trial follow-up appointments, such as childcare, time off work, travel, transportation and associated financial costs to fulfil trial data collection requirements, were cited as problematic^1225 27^,^30^:

Draper *et al*^28^ [page 6 ] (first order construct): “…I stay with my kids and they are still young, so for me coming here [clinic visit], I had to get someone to look after my kids, and sometimes you find that I tell a person that I am going to be gone for so long, but I end up taking much longer time here, which will then prevent the person to help me the next time when I ask for help”. (Withdrawn participant—non-intervention) (clinic visits).

Participants also struggled with the personal time commitments required to maintain participation in a trial and were cited as reasons for missed clinic visits^27^,^2931^ or not returning questionnaires^12 30^:

Henshall *et al*^29^ [page 6] (first order construct): “I left… Because I didn’t have enough time… The study is of such a long duration and I just found that too challenging with work…” EXTOD 17 (male, Bristol) (clinic visits).

Authors in one study^25^ reported that the personal burdens of trial participation were exacerbated for many participants as they struggled to balance low-paid jobs demanding long hours with attending clinic visits for data collection purposes. One participant highlighted the challenges of having a job which caused her to miss *“five consecutive clinic visits because of her demanding working hours and infrequent time off” (mid 20s, FGD)*^25^*[5] (second order construct) (clinic visits).*

#### Subtheme 2.2: type and timing of data collection

The type and timing of data collection was reported by participants as an important consideration when completing outcome assessments. In particular, questionnaires were cited as burdensome,^11 12 29 30^ being described as “*too long and time consuming*”^30^[229] (*second order construct*), *“pretty boring”*^11^*[5] (Participant 3)*, or *“a bit monotonous” and “very repetitive”*^12^*[6] (online supplemental file) (Female, late 30s, CGALL study)* ultimately leading to non-completion.

To support data collection, some participants expressed preferences for completing a shorter version of the questionnaire on the telephone with a trial team member, rather than self-completion and returning by post.^11 12^

The timing of data collection (including frequency of data collection) was reported as influencing compliance with data completion.^11 29^ If data collection requirements coincided with a time that was perceived as inconvenient by participants, they could be less inclined to engage with trial process requirements.^29^

Added to this, there was also the issue for some participants of potentially being unable to recall information accurately over time, which could impact completion of outcome assessments.^11^

Factors relevant to influencing trials processes (but outside control of the trial team) could also be linked to timing of data collection and its influence on retention, for example, a postal strike was cited by one participant as a reason for non-completion of a questionnaire.^30^

#### Strategies to support participants in overcoming trial burdens related to data collection

Participants reported the use of strategies which largely revolved around themselves and/or family members^11 25^ to help them fulfil data collection requirements:

Magazi *et al*^25^ [page 4] (first order construct): “He[partner] fetched me from my house…he asked me why didn’t I go [to the trial clinic] because they [study staff] said I missed my visit. I said my phone is not working. So, he [partner] brought me here [to the trial clinic]” (age 18-21, FGD) (clinic visits).

Other strategies described to support follow-up data collection included how trial teams did (or could have) encouraged questionnaire return, such as using reminders and monetary incentives.^11 12 30^ For example, in the Lawrie *et al* study,^11^ preferences for additional support from the trial team to support completion and return of questionnaires were reported.

Where a trial team did use strategies such as providing participants with self-addressed envelopes^12^ or reminders to prompt questionnaire return,^11^ it was considered helpful. It was also suggested in the Newlands *et al* study^12^ that using a ‘buddy’ approach to support participants’ ongoing engagement in a trial could be a potentially helpful strategy. Another study^28^ reported that providing transport for trial participants to attend a clinic visit was considered helpful.

Using a monetary strategy to incentivise data collection among participants was generally reported as not expected or favourably regarded, as participants described not wanting to feel ‘bribed’^11 12^:

Newlands *et al*^12^ [page 9 online supplemental file] (first order construct): “No reward or incentives would have (been) required. I think if you’re going to do these things then do them willingly” (Female, late 50’s, DISCO Study) (postal questionnaire).

However, authors in two studies reported how the use of incentives could potentially have encouraged some participants to respond better to questionnaire completion,^30^ and the lack of ‘*pressure’* from a trial to encourage participant questionnaire completion was a potential barrier to incentivising participants.^11^

An additional aspect of strategies to support data collection was highlighted in two studies, whereby organisational aspects relating to follow-up outcome assessment were reported as problematic for trial retention.^12 27^ In particular, participants’ perceptions of a trial team’s inflexibility to accommodate participant attendance at follow-up clinic visits, or poor organisation surrounding clinic visits and *“service-related logistical issues”*^27^*[467] (second order construct)* were cited as reasons for non-attendance at follow-up clinics for outcome assessments^12 27^:

Newlands *et al*^12^ [page 2 online supplemental file ] (first order construct): “Yeah, they did invite me for a clinic appointment …but due to work commitments I wasn’t able to make those slots. So, I gave them a call and asked if we could rearrange different slots and they weren’t able to do that, so I wasn’t able to complete the study.” (Female, mid 30’s, INTERVAL study) (clinic visits).

### Theme 3: navigating life as a trial participant

This theme highlights the wider context in which people were trial participants, concurrent to navigating their everyday lives, that were not directly related to trial participation.^1225 27 28 30^,^32^ Findings describe three subthemes: 1. life gets in the way; 2. perceptions of self; 3. cultural community context and how these issues impacted trial non-retention.

#### Life gets in the way

The importance of the wider context in which people participated in trials could influence non-retention. Examples of life getting in the way for trial participants included issues such as work commitments,^28 31^ moving house, changing priorities,^31^ as well as *“pregnancy, exams”*^30^*[229] (second order construct),* college commitments*,*^25^ *“ill family” member*^32^*[8](second order construct),* involvement in another study,^30^ and other health-related treatment commitments.^27^ Authors in the Draper *et al* study^28^ highlighted how some of these issues could be made worse by a lack of social support which could negatively influence a participant’s ability to stay involved in a trial:

Draper et al^28^ [page 4] (second order construct): “The absence of social support was mentioned by some participants, which negatively influenced their ability to stay involved in the trial, and are therefore relevant to retention. This was to do with not having someone to take care of their other child/children when attending the research site (or not being able to pay someone to look after their child), expectations of family members to receive some or all of the financial incentive that participants receive…” (clinic visits)*.*

While most participants reported life events and commitments could interfere with trial participation to some extent, one participant highlighted their changing circumstances of becoming a carer during the trial had not interfered with their commitment to trial participation. However, this outlook and commitment was ultimately insufficient to ensure they remained a trial participant.

#### Perceptions of self

For some participants, their reported self-perceptions were acknowledged as undermining their engagement with trial data collection. For example, some participants described personal habits such as laziness^30 31^ being *“forgetful, disorganised”*^30^[229] *(first order constructs)* and having *“issues with procrastination”*^31^*[10]* (*first order construct*), as reasons for failure to complete data collection, at various time points.

Furthermore, a perception of fear towards incorrectly answering questions was highlighted by one participant as a reason for questionnaire non-response, while anxiety about attending the dentist was cited by another participant as a reason for a missed clinic visit (dental related)^12^:

Newlands *et al*,^12^ [online supplemental file, page 6] (first order construct)” It’s kind of like, “What’s this person going to think of me? Am I answering it right?” (Female, late 50’s, DISCO Study)” (postal questionnaire).

#### Cultural community context

For some trial participants, their cultural settings shaped their abilities to fulfil outcome assessment data collection requirements.^25 27 28^ The Magazi *et al*^25^ and Draper *et al*^28^ studies were both set in low-income townships in Johannesburg, South Africa, and the Wells study^27^ in Latina, Spanish-speaking, low-income communities based in the USA. Within these communities, resource constraints, cultural norms and family/gender-based social influences were cited as contributing factors to non-retention.

Female participants specifically reported the influence of male family members and how this could impact being unable to complete data collection requirements.^25^ For example, a young participant described how her father had *“forced her”*^25^*[4] (age 18-21y, EI) (first order construct)* to visit home regularly, which resulted in numerous missed clinic appointments. Other reported cultural considerations included bereavement traditions, as one woman described multiple missed clinic visits due to her husband’s funeral, the length of mourning period and cleansing such rituals associated with such an event.^25^ In another study, authors outlined cultural issues such as, *“language communication problems”* and *“discrimination from providers.”*^27^[467] *(second order construct)* as reasons participants withdrew from a trial.^27^

Authors in the Draper *et al*^28^ study also highlighted the importance of family and community member perceptions related to aspects of trial participation, which could negatively impact continued trial engagement:

Draper *et al*^28^ [page 4 ] (first order construct) :“ “Because I felt like when I speak to my family members, like they were judging me or wanted to tell me how to go about doing things, even if I wanted to do the things the way I wanted to, so that I can make my own mistakes, learn from my mistakes. (Withdrawn participant—non-intervention) (clinic visits).

### Theme 4: managing expectations of participation

This fourth theme highlights the interplay between participants’ expectations of trial participation and their actual experiences, and how this can impact trial non-retention.^11 12 25 27 28 30^ Findings include two subthemes related to: (1) the sense of care and relational support provided through trial participation and (2) awareness of what is expected as a trial participant, and how these impacted trial non-retention.

#### Subtheme 4.1: care and relational support offered by trial participation

Not meeting expectations of care and relational support from trial staff was reported by participants as being influential on non-completion of trial outcome data, across a number of studies.^11 12 25 27 28^ Expectations such as receiving greater medical attention, feeling supported and valued by trial staff were reported as important to participants—but were not always fulfilled^11 12^:

Lawrie *et al*^11^ [page 3] (second order construct): “participants suggested that activities in the trial did not meet their initial expectations, with some indicting that they expected a greater level of medical attention…” (postal questionnaires).

Newlands *et al*^12^ [page 1 online supplemental file ] (first order construct): “There was no care involved, it was just two questionnaires”. (Female, mid 30’s, INTERVAL Study) (postal questionnaires).

A perceived lack of responsiveness from trial staff, was also highlighted in two studies,^12 28^ as a factor negatively impacting participants’ continued engagement in a trial:

Draper *et al*^28^ [page] (first order construct) :“The lady that recruited me had said that she would call me, but she didn’t call me. She called me after some months after I had not been here and she wanted to know if I was no longer interested in the study, I told her that I was interested but I wasn’t getting any communication from her, and she said that she had been calling me but was not getting through, so maybe I had not charged the phone”. (Withdrawn participant—non-intervention) (clinic visits).

Newlands *et al*^12^ [page 5 online supplemental file ] (first order construct): “ “I wrote a letter, a little note alongside it [questionnaire] and popped that in and said I’d welcome a telephone call or a conversation about the study at that point, and I received no reply. So to be truthful I lost a bit of interest at that point.” (Male, mid 70’s, MASTER study) (postal questionnaire).

Problematic *“patient-provider problems”*^27^*[467] (second order construct)* were also highlighted by some participants as resulting in missed clinic visits and impacting retention^12^:

Newlands *et al*^12^ [page 3 online supplemental file ] (first order construct): “Yeah, I felt a bit anxious about if the nurse was going to be mean to me again. I didn’t look forward to going to the visits.” (Female, mid 40’s,) (clinic visits).

#### Subtheme 4.2: understanding what is expected as a trial participant

Across multiple studies,^11 12 27 30^ participants’ understanding of what was expected of them relating to completion of follow-up outcome assessments appeared ambiguous. This resulted in confusion for some participants, particularly if they had completed some (but not all) trial follow-up assessments and had not articulated a deliberate wish to leave a trial.

Labelling participants as trial ‘drop outs’, even though they had completed some (but not all) trial follow-up assessments was highlighted in the Wells *et al*^27^ study and illustrated the misunderstanding and confusion that existed:

Wells *et al*^27^ [page 468] (first order construct):“It wasn’t dropping out because I didn’t not want to get help…It’s not like it was under my power. You know what I mean? You can say [dropout] if you want to…but I don’t feel like I want[ed] to get out of the study.” (clinic visits).

Likewise, authors in the Nakash *et al*^30^ study highlighted how participants’ lack of awareness about compliance with trial follow requirements was more likely to impact non-retention than anything they specifically disliked about the trial:

“Nakash *et al*^30^ [page 229] (authors’ interpretation): “ The majority of participants who failed to respond were keen to stress that their lack of response was through no fault of the trial itself and they were happy to continue to be involved.” (postal questionnaires).

Confusion about the trial’s duration was also identified in one study^11^ as adding to uncertainties about participants’ expectations of trial involvement:

Lawrie *et al*^11^[page 3] (second order construct): “The majority of participants reported being unaware of the duration of their participation within the trial…” (postal questionnaires).

A lack of awareness about the importance of complying with trial follow-up outcome assessments was illustrated in the Newlands *et al* study,^12^ where some participants questioned the value of their ‘small’ contribution and the difference it would make to the trial:

Newlands *et al*^12^ [page 2 online supplemental file] (first order construct): “No, I didn’t think I would make much difference at all, to be honest. I was very doubtful about the value of it (attending a clinic appointment)… .Well, it’s just a small contribution that would probably add up to something at the end of the day, but it was a very small contribution. (Female, 85-90yrs, INTERVAL study) (clinic visits).

In the Lawrie study,^11^ some participants described a sense of ‘duty’ to complete follow-up questionnaires, and ‘guilt’ if they failed to return them. However, these feelings were not enough to keep them in the trial.

## Discussion

This review has synthesised published evidence on participant-reported factors that can influence non-retention within RCTs. We identified 11 studies (reporting qualitative data from 14 separate trials) that included participant-reported reasons for not completing components of trial processes related to data collection for outcome measures.

This review has identified that participants’ fluctuating health, balancing of trial burdens, life circumstances and trial expectations were interconnected by an overarching theme of ‘push and pull’ influences, reflecting a fluidity of factors impacting trial non-retention.

The findings demonstrate that retention is an ongoing process, ebbing and flowing across the trial timeline. The studies included highlight the importance of a nuanced understanding of how trial non-retention may be linked to inconsistencies in participants’ completion of follow-up outcome assessments, which can at times result in unintentional dropout. For most participants, this was shaped by their understanding of trial expectations (at recruitment and beyond), particularly follow-up outcome assessments, and by their expectations of trial participation, including perceived benefits. However, it is important to note that most included studies did not report the perspectives of participants who had actively withdrawn from a trial.

Concerns about ensuring potential trial participants are informed at the time of recruitment and what is expected of them in terms of ‘full participation’ were reported previously by Skea *et al*.^10^ However, our synthesis provides more in-depth understanding of how these influences can specifically impact on return of data or attendance at follow-up visits, either deliberately or by default, and potential aspects that may be modifiable to improve retention.^34^,^36^ Our synthesis highlights a nuanced understanding of why participants discontinued data collection, or aspects of it, particularly when they perceived no personal benefit or felt recovered, and therefore perceived little value in continuing.

Our findings also highlight the importance of understanding the cultural settings for some trials, where male partners, family members, community elders or societal traditions can have significant influence on trial non-retention, particularly for female trial participants. This is an important finding and one that has not been given much attention in the literature specific to the collection of trial outcome data. While this is important, it should be set in the context of understanding what may (or may not) be modifiable in terms of supporting trial retention in culturally diverse settings. We know from current efforts the drive there is on increasing diversity within clinical trials.^37 38^ However, much of this focus remains on trial recruitment and as we have set out previously, trial design cannot lose sight of the interconnectedness of recruitment and retention, and the strategies needed to support both,^39^ including patient partner involvement at trial design stage to better understand and support data collection strategies to improve retention.

While our synthesis has reported a range of issues contributing to non-retention related to collection of outcome data, some of which may be challenging to modify, improving communication at the point of trial consent may help anticipate and mitigate some of these issues. Clearly explaining the rationale for continued engagement with data collection, and openly discussing how common issues may deter participants from completing follow-up outcome data collection, offers an opportunity to address these concerns early in the trial process.

Although certain aspects of trial design may influence how participants perceive the relevance of engaging with outcome data collection, the evidence presents a mixed picture, with influences operating at multiple levels. Factors such as clinical specialty, the acute or chronic nature of the condition under study, intervention complexity, multimorbidity and the relational dynamics between participants and site staff may each shape, to varying degrees, participants’ engagement with completing outcome measures. Although trial interventions were not the focus of this review, the type of intervention could also potentially influence how participants engage with completing outcome data collection.

Recent research has shown that the topic of retention is not commonly discussed in trial recruitment consultations, and if it is included, the information can often be inaccurate, for example, in relation to the timing and frequency of follow-up questionnaires.^34^ Retention is rarely addressed in participant information leaflets,^35 36^ providing important context for our findings on participants’ lack of awareness of follow-up requirements. Although trial teams may be concerned that providing participants with too much information could negatively impact recruitment, it could be argued that setting clearer expectations of participation may contribute to improved retention. Further, in the recent Cochrane review on factors impacting on recruitment to trials,^40^ the authors reported the importance of how trial information is communicated to potential participants including expectations such as time commitments. We also know from previous research that people often decide to take part in a trial in the hope of personally benefitting in some way from an intervention, although reporting they are doing so for altruistic reasons—so-called ‘conditional altruism.’^41^

As such, potentially differing expectations of engagement with data collection reinforce the need for clear, tailored pretrial explanations about what data will be collected, why it is important, and the expected time and effort involved. Ensuring participants have a realistic understanding of these requirements may not only support more informed decision making, but also support more complete outcome data collection.

More broadly, the influences we have identified in this synthesis and how these link to the recent Cochrane review^42^ examining strategies to improve trial retention suggest a mismatch between trialists’ ‘traditional’ retention approaches (eg, monetary incentives) and what participants report as salient for their retention. Our synthesis highlights that trial participants often regard monetary incentives ambivalently, viewing them as unnecessary or inappropriate since they are not the primary motivation for ongoing trial participation. Involving patient public partners in the decision-making process regarding retention strategies or their involvement within the strategy itself may prove beneficial for trial teams when choosing a retention strategy. For example, in the Gillies *et al*’s review,^42^ one study demonstrated a 21% increase in retention by using a retention strategy whereby peer researchers (other trial participants) telephoned participants to encourage or collect follow-up data collection.^43^ Although we are not specifically advocating this specific retention strategy, it does provide useful insight into drivers of retention for some trial participants.

Our synthesis also identified the importance of timing of trial information, particularly for potential participants receiving a new health diagnosis, and how this could impact outcome data collection. Although early diagnosis and early intervention have been linked with improved clinical outcomes in many disease areas, our synthesis highlighted how a recent health diagnosis can present challenges for trial retention. Issues such as heightened emotional distress, uncertainty or information overload can make ongoing completion outcome data collection more difficult. As such, non-completion of outcome assessment data may be a particular risk for these participants, highlighting the need to address such issues during trial consent discussions.

From an ethical perspective, issues related to trial non-completion raise important questions about how to balance participants’ right to withdraw voluntarily with the responsibility to ensure participation decisions are well-informed and not perceived as potentially coercive. While it is essential that trial participation remains voluntary, providing clear information about what trial involvement entails, including expectations about outcome data collection, would support more informed decision making and lessen ethical tensions. These issues highlight the need for future research to explore how best to communicate expectations without exerting undue influence, ensuring that decisions remain both fully informed and voluntary.

In this synthesis, recognising that trial participants may experience both ‘push and pull’ influences simultaneously or shift between them over time offers trialists an opportunity to anticipate and consider ways to support informed and sustained trial participation, specific to completion of follow-up outcome assessments. Advances in digital technology may create additional opportunities to support participants with follow-up data collection, and the implications of these developments for trial design are important to consider. However, any proposed solutions require careful exploration at the design stage to ensure approaches remain methodologically robust, ethically sound and acceptable to participants and do not introduce unintended consequences, for example, a remote method of data collection could negatively impact on retention if participants are motivated by seeing the clinician in a hospital for follow-up.

The studies included in this review were primarily conducted in the UK; however, there was representation of trial participants from low-middle-income settings in South Africa^25 28^ and the USA.^27^ Including perspectives from ethnic minority and lower socioeconomic groups is important for understanding trial non-retention issues and developing culturally sensitive strategies to support trial retention, linked to supporting outcome data collection.

Using the GRADE-CERQual Assessment of Confidence in the Evidence, the ‘moderate confidence’ across findings indicates that the review team considers that it is likely that the review finding is a reasonable representation of the phenomenon of interest, although with some concerns, particularly regarding the adequacy of the data relating to the richness and quantity presented in some studies. Using the GRADE-CERQual approach to provide a transparent assessment of confidence in the review findings may help trialists identify more easily which areas to prioritise for potential intervention development to support trial retention.

### Strengths and limitations

In this review, we have undertaken a comprehensive and systematic literature search to identify studies providing qualitative data on trial participants’ reasons for trial non-completion linked to trial processes ie, data collection for outcome measures across a range of diverse trial contexts. To our knowledge, this is the first study to synthesise primary qualitative data exclusively on participants’ reasons for non-completion of data collection of outcome measures. A key strength of this review is the up-to-date search which was conducted through to February 2025. This strengthens the findings as they capture and reflect the most recently published literature on the topic. The review includes studies that span across a wide range of clinical contexts, which facilitates the transferability of findings. Although studies included in this review were predominately from the UK, a strength was the inclusion of three studies conducted in diverse low-income community settings.

We used the CASP checklist for quality assessment of the included papers and, through the application of GRADE CERQual, we identified a level of confidence in each of our key findings. Although there was variability in the quality of some of the included studies, we did not exclude on this basis, due to the small number of studies included and their contribution to the synthesis. It is possible that potentially eligible papers were missed during searching/screening stages, but this risk was minimised by using multiple review team members to support these review stages. Another limitation relates to the richness of the data; some of the studies provided thin and insufficiently rich data to fully support the findings.

Furthermore, in some instances, studies reported reasons for both non-retention due to the trial intervention and non-retention due to trial processes, that is, data collection. In these cases, it was challenging to disentangle the data and extract data specifically related to non-retention due to trial processes. This was a particular challenge when trying to characterise participants who were non-retainers exclusively due to trial processes. While we have included three studies that are conducted in diverse low-income community contexts, the majority of studies represent trials conducted in high-income settings. As such, the findings may not be transferable to all healthcare settings and participant populations. Finally, we were unable to undertake PPI activity for this review due to the absence of dedicated funding or resources to support meaningful involvement. We acknowledge this as a limitation and would plan, in future updates of the review, to secure the necessary resources to enable PPI input.

### Implications for research

It is widely recognised that variations such as healthcare settings, trial types and clinical conditions may shape retention in different ways. Our synthesis has illustrated how ‘push and pull’ influences can shape participants’ engagement with trial data collection across the trial timelines, reflecting the fluid interplay of fluctuating health, trial expectations, perceived benefits and broader life circumstances. Understanding of these issues and how they can contribute to trial non-retention is an important step towards providing trialists with information needed to potentially modify aspects of trial design and develop targeted interventions to support retention efforts going forward.

### Conclusions

This QES is the first to have synthesised primary qualitative evidence on participants’ reasons for trial non-completion, focusing specifically on non-retention linked to outcome data collection. This review has generated important new insights that extend beyond individual studies, identifying an overarching theme of a ‘push and pull’ dynamic that describes the shifting influences over time on trial participants’ engagement with outcome data collection. The evidence highlights the need for further research on how to better support trial recruitment discussions that set out clear and realistic expectations to potential trial participants, and on strategies that recognise, and where possible address, some of the influences on participants to improve outcome data completeness and ultimately improve trial retention.

## Supplementary material

10.1136/bmjopen-2025-111824 online supplemental file 1

10.1136/bmjopen-2025-111824 online supplemental file 2

10.1136/bmjopen-2025-111824 online supplemental file 3

10.1136/bmjopen-2025-111824 online supplemental file 4

10.1136/bmjopen-2025-111824 online supplemental file 5

10.1136/bmjopen-2025-111824 online supplemental file 6

10.1136/bmjopen-2025-111824 online supplemental file 7

## Data Availability

All data relevant to the study are included in the article or uploaded as supplementary information.
