# Supplementary material for: Retention in trials: a qualitative evidence synthesis of studies reporting participant reasons for trial non-completion
Source: BMJ Open. 2026 Apr 20;16(4):e111824. doi: 10.1136/bmjopen-2025-111824 (PMC13110579; doi:10.1136/bmjopen-2025-111824)
Supplement: online supplemental file 2 [file bmjopen-16-4-s002.docx]

**GRADE-CERQual Table**

| **Summary of Review Findings** | **Studies contributing to the Review Finding** | **Methodological limitations** | **Coherence** | **Adequacy** | **Relevance** | **Overall GRADE-CERQual Assessment of Confidence in the Evidence** | **Explanation of CERQual Assessment** |
| --- | --- | --- | --- | --- | --- | --- | --- |
| **Theme 1: Fluctuating health** | Henshall, Kehagia Wells, Nakash, Lawrie, Nicholas, Postel, Sanders |  |  |  |  |  |  |
| **1.1 Coming to terms with a diagnosis**  Some participants struggled with the timing of recent health diagnosis/condition concurrent to trial participation. Feelings of overwhelm, being confronted and the emotional impact of dealing with a diagnosis/condition was reported as making it especially difficult for some people to engage with trial information. Disruption to routine care and health service provision was also reported as impacting on non-retention. | Henshall, Wells, Nicholas, Sanders | Minor concerns  All four studies were  assessed as having minor methodological limitations | No concerns. Variations in the data reported reflecting a good fit between data in primary studies with review finding. | Moderate concerns  There was a lack of richness in the data  reported across the 4 studies, with limited details to gain an in-depth understanding of the issues. | Moderate concerns. Studies represented trial setting  experiences in the UK, Australia, and USA (Latina context), thus limited geographical spread.  The characteristics of participants were provided in all studies, however, the characteristics (age, gender, ethnicity etc) of those who were not retained were not always clearly reported in studies where both retained and non-retained participants were included  The included 4 studies encompassed a limited range of clinical settings (diabetes & mental health). Data collection across studies was clinic visits or online completion of data collection | Moderate confidence | Minor concerns about methodological limitations. Moderate concerns about adequacy and relevance. |
| **1.2 Changeability to health**  Participants reported aspects of changes to their health (positive and negative) during trial engagement. Changeability was reported as influencing trial non-retention related to follow up outcome data collection, e.g. not seeing relevancy of completing a follow up questionnaire if feeling their health had improved. | Kehagia Nakash, Lawrie, Nicholas, Postel | Minor concerns  All five studies were  assessed as having minor methodological limitations | No concerns.  Variations in the data reported, reflecting a good fit between data in primary studies with review finding. | Moderate concerns.  There was a lack of richness in the data  reported across 4 studies, with limited details to gain an in-depth understanding of the issues. One study provided slightly richer data but still relatively thin. | Minor concerns.  Studies represented trial setting  experiences in the UK, Australia, and Europe, thus contextually not inclusive of middle-low-income settings.  The characteristics of participants were provided in all studies, however, the characteristics (age, gender, ethnicity etc) of those who were not retained were not always clearly reported in studies where both retained and non-retained participants were included.  The included 5 studies encompassed a range of clinical conditions across 5 individual trials. Data collection ranged from clinic visits, postal questionnaires and online data collection. | Moderate confidence | Minor concerns about methodological limitations and relevancy. Moderate concerns regarding adequacy. |
| **Theme 2: Balancing trial burdens** | Henshall, Magazi, Newlands, Wells, Nakash, Lawrie, Draper |  |  |  |  |  |  |
| **2.1 Personal costs associated with trial participation** Participants reported the burdensome nature of trial participation in terms of personal opportunity costs directly linked to participating in a trial. Issues such as childcare, time off work and travel costs were specifically reported as influencing trial non-retention. These issues were exacerbated for participants in low middle income countries. | Henshall, Magazi, Newlands, Wells, Nakash, Draper | Minor concerns.  All six studies were  assessed as having minor methodological limitations | Minor concerns.  Data reported reflecting a good fit between data in primary studies with review finding. However,  the issue of costs was difficult to disentangle e.g. exact personal cost related trial participation e.g. clinic visit, rather than life gets in the way type issues. | Moderate concerns.  There was a lack of richness in the data  reported across the studies, with limited details to gain an in-depth understanding of the issues. | No concerns.  Studies represented trial setting  experiences mainly in the UK. The other two studies were set in the USA (Latina context), and South Africa.  . The inclusion of the studies set in South Africa (Magazi and Draper) and USA Latina community (Wells) provided additional understanding of contextual relevancy influencing non-retention.  The included 6 studies encompassed a range of clinical settings. 5 studies reported on recruitment of participants from a single trial, with 1 study (Newlands) reporting on participant recruitment across 5 different clinical trials. Data collection included clinic visits and postal questionnaires  The characteristics of participants were provided in all studies, however, the characteristics (age, gender, ethnicity etc) of those who were not retained were not always clearly reported in studies where both retained and non-retained participants were included. | Moderate confidence | Minor concerns about methodological limitations and coherence. Moderate concerns about adequacy. |
| **2.2 Type and timing of data collection**  Participants reported barriers to data collection specifically related to type and timing of data requirements, which impacted trial retention.  The majority of participants reported on data collection linked to follow up questionnaires. | Henshall, Newlands, Nakash, Lawrie | Minor concerns.  All four studies were  assessed as having minor methodological limitations | No concerns.  A good fit between data in primary studies with review finding. | Moderate concerns.  There was a lack of data richness, with most of the data reported based on findings from two studies (Lawrie and Newlands). Although these 2 studies offered more descriptive data than the other two studies, they were relatively limited in the depth of data reported. | Moderate concerns.  The 4 included studies only represented UK trial settings.  .  3 studies reported on recruitment of participants from a single trial, with 1 study (Newlands) reporting on issues across 5 different clinical trials. Data collection included clinic visits and postal questionnaires.  The characteristics of participants were provided in all studies, however, the characteristics (age, gender, ethnicity etc) of those who were not retained were not always clearly reported in studies where both retained and non-retained participants were included. | Moderate confidence | Minor concerns about methodological limitations. Moderate concerns about adequacy and relevance. |
| **2.3 Strategies to support continued participation**  Participants reported their personal attempts to support completion of outcome data assessments as well as attempts by trial teams. Although strategies were reported as facilitating some participants at various stages, ultimately, attempts to support trial retention were not successful. | Lawrie, Magazi, Newlands, Nakash, Wells. Draper | Minor concerns.  All six studies were  assessed as having minor methodological limitations. | No concerns.  The data reported reflects a good fit between data in primary studies with review finding. | Moderate concerns.  None of the six studies provided rich data to support a deep understanding of the issues. Two studies (Lawrie and Newlands) provided most of the data for this sub-theme. | Minor concerns  The six included studies represented trial settings in the UK, USA (Latina context) and S. Africa  .  The inclusion of the study sets in South Africa (Magazi and Draper) and the Latina community in the USA (Wells) provided additional contextual understanding of attempts to support retention.  Five studies reported on recruitment of participants from a single trial, with 1 study (Newlands) reporting on issues across 5 different clinical trials. Data collection included clinic visits and postal questionnaires.  The characteristics of participants were provided in all studies, however, the characteristics (age, gender, ethnicity etc) of those who were not retained were not always clearly reported in studies where both retained and non-retained participants were included. | Moderate confidence | Minor concerns about methodological limitations and relevance. Moderate concerns about adequacy. |
| **Theme 3: Navigating life as a trial participant** | Magazi, Newlands, Wells, Nakash, Nicholas, Postel, Draper |  |  |  |  |  |  |
| **3.1 Life gets in the way**  Participants shifting life priorities and commitments outside of the trial e.g. moving house, college and pregnancy, interfered with ongoing trial participation and completion of outcome assessments. | Nicholas, Nakash, Magazi, Postel, Wells, Newlands, Draper | Minor concerns.  All seven studies were  assessed as having minor methodological limitations. | No concerns.  The data reported reflects a good fit between data in primary studies with review finding. | Moderate concerns.  None of the seven studies provided rich data to support a deep understanding of the issues. One study (Newlands) provided most of the data for this sub-theme, albeit thin data. | No concerns.  The 7 included studies represented a broad range of trial settings in the UK, USA (Latina context), S. Africa, Netherlands, Australia.  The inclusion participants in South Africa (Magazi and Draper) and the Latina community in the USA (Wells), provided additional contextual understanding of these issues.  6 studies reported on recruitment of participants from a single trial, with 1 study (Newlands) reporting on issues across 5 different clinical trials. Data collection included clinic visits, postal questionnaires and online data collection.  The characteristics of participants were provided in all studies, however, the characteristics (age, gender, ethnicity etc) of those who were not retained were not always clearly reported in studies where both retained and non-retained participants were included. | Moderate confidence | Minor concerns about methodological limitations. Moderate concerns about adequacy. |
| **3.2 Perceptions of self**  Participants own perceptions of themselves, and their capabilities can potentially undermine the completion of data collection activities. Personal feelings of anxiety and fear over aspects of completing data collection was also cited as a barrier to follow-up completion. | Nakash, Nicholas, Newlands, | Minor concerns.  All three studies were  assessed as having minor methodological limitations. | No concerns.  The data reported reflects a good fit between data in primary studies with review finding. | Moderate concerns.  None of the three studies provided rich data to support a deep understanding of the issues. One study (Newlands) provided most of the data for this sub-theme, albeit thin data. | Moderate concerns.  The 3 included studies represented a narrow range of geographical trial settings (UK; Australia).  The 3 studies described relevancy, including a range of clinical contexts and types of data collection e.g. clinic visits, questionnaires, online programme.  The characteristics of participants were provided in all studies, however, the characteristics (age, gender, ethnicity etc) of those who were not retained were not always clearly reported in studies where both retained and non-retained participants were included. | Moderate confidence | Minor concerns about methodological limitations. Moderate concerns about adequacy and relevance. |
| **3.3 Cultural community context**  Cultural context and the impact of community and family influenced non-retention in some trials in low-income settings. | Magazi, Wells, Draper | Minor concerns  All three studies were  assessed as having minor methodological limitations | Minor concerns.  The data reported reflects a good fit between data in primary studies with review finding. However, extrapolating data specific to trial non-retainers was problematic. | Moderate Concerns  None of the three studies provided rich data to support a deep understanding of the issues. Data reported in the 3 studies is very thin with limited information about the issues. | Moderate concerns.  The 3 included studies represented a narrow range of geographical trial settings (South Africia and USA Latina context)  The studies focused on outcome data collection via clinic visits only and the clinical contexts limited to HIV (Magazi), maternal health (Draper) and mental health (Wells).  In the three studies, participants were limited to females only, but this provided some understanding of how cultural contexts can impact women in terms of trial participation. | Moderate Confidence | Moderate concerns regarding adequacy of data. Moderate concerns about relevance. |
| **Theme 4: Managing expectations of participation** | Magazi, Newlands, Wells, Nakash, Lawrie, Draper |  |  |  |  |  |  |
| **4.1 Expectations of care and relational support offered by trial participation**  Not meeting participants expectations regarding care and support from trial staff, and participants feeling their participation in the trial is not valued or acknowledged, reported as impacting on trial non-retention. | Magazi, Newlands, Wells, Lawrie, Draper | Minor concerns  All five studies were  assessed as having minor methodological limitations. | No concerns  The data reported reflects a good fit between data in primary studies with review finding. | Moderate concerns.  There was a lack of richness in the data  reported across the 5 studies, with limited details to gain an in-depth understanding of the issues.  Most of the data for this theme was from one study (Newlands).  . | No, minor concerns.  The 5 included studies included trials in UK, in Africa and in USA (Latina context).  The 5 studies included a wide range of clinical contexts.  The inclusion participants in South Africa (Magazi, Draper) and the Latina community in the USA (Wells), provided additional contextual understanding of these issues.  4 studies reported on recruitment of participants from a single trial, with 1 study (Newlands) reporting on issues across 5 different clinical trials.  Data collection focused on postal questionnaires and clinic visits.  The characteristics of participants were provided in all studies, however, the characteristics (age, gender, ethnicity etc) of those who were not retained were not always clearly reported in studies where both retained and non-retained participants were included. | Moderate confidence | Minor concerns about methodological limitations and relevancy. Moderate concerns with adequacy. |
| **4.2 Understanding what is expected as a trial participant** Participants often were unclear about follow-up requirements, the implications of not completing follow-up, and questioning their personal contribution  and value to the study. Some participants reported confusion about being labelled as a ‘drop-out’. | Wells, Nakash, Lawrie, Newlands | Minor concerns.  All four studies were  assessed as having minor methodological limitations | No or very minor concerns.  The data reported reflects a good fit between data in primary studies with review finding. | Moderate concerns.  There was a lack of richness in the data  reported across the 4 studies, with limited details to gain an in-depth understanding of the issues. | Minor concerns.  The 4 included studies included trials in the UK and in the USA (Latina context).  The 4 studies included a wide range of clinical contexts and data collection focussed on clinic visits and questionnaires.  Participants in one study (Wells) were limited to females only. | Moderate confidence. | Minor concerns regarding methodological limitations, coherence and relevance. Moderate concerns with adequacy. |

References

1. Nakash RA, Hutton JL, Lamb SE, Gates S, Fisher J. Response and non‐response to postal questionnaire follow‐up in a clinical trial–a qualitative study of the patient’s perspective. Journal of evaluation in clinical practice. 2008;14(2):226-35.

2. Nicholas J, Proudfoot J, Parker G, Gillis I, Burckhardt R, Manicavasagar V, et al. The ins and outs of an online bipolar education program: a study of program attrition. Journal of Medical Internet Research. 2010;12(5):e57.

3. Wells AA, Palinkas LA, Qiu X, Ell K. Cancer patients’ perspectives on discontinuing depression treatment: the “drop out” phenomenon. Patient preference and adherence. 2011:465-70.

4. Postel MG, de Haan HA, Ter Huurne ED, Becker ES, de Jong CA. Effectiveness of a web-based intervention for problem drinkers and reasons for dropout: randomized controlled trial. Journal of medical Internet research. 2010;12(4):e1642.

5. Sanders C, Rogers A, Bowen R, Bower P, Hirani S, Cartwright M, et al. Exploring barriers to participation and adoption of telehealth and telecare within the Whole System Demonstrator trial: a qualitative study. BMC health services research. 2012;12(1):1-12.

6. Henshall C, Narendran P, Andrews RC, Daley A, Stokes KA, Kennedy A, et al. Qualitative study of barriers to clinical trial retention in adults with recently diagnosed type 1 diabetes. BMJ open. 2018;8(7).

7. Magazi B, Stadler J, Delany-Moretlwe S, Montgomery E, Mathebula F, Hartmann M, et al. Influences on visit retention in clinical trials: insights from qualitative research during the VOICE trial in Johannesburg, South Africa. BMC women's health. 2014;14(1):1-8.

8. Lawrie L, Duncan EM, Dunsmore J, Newlands R, Gillies K. Using a behavioural approach to explore the factors that affect questionnaire return within a clinical trial: a qualitative study based on the theoretical domains framework. BMJ open. 2021;11(4):e048128.

9. Newlands R, Duncan E, Presseau J, Treweek S, Lawrie L, Bower P, et al. Why trials lose participants: a multitrial investigation of participants’ perspectives using the theoretical domains framework. Journal of clinical epidemiology. 2021;137:1-13.

10. Kehagia AA, North TK, Grose J, Jeffery AN, Cocking L, Chapman R, et al. Enhancing trial delivery in Parkinson’s disease: Qualitative insights from PD STAT. Journal of Parkinson's Disease. 2022;12(5):1591-604.

11. Draper CE, Tshetu N, Nkosi N, Lye S, Norris SA. Retention in the Bukhali trial in Soweto, South Africa: a qualitative analysis using self-determination theory. BMJ Global Health. 2025;10(2).
