# Supplementary material for: Retention in trials: a qualitative evidence synthesis of studies reporting participant reasons for trial non-completion
Source: BMJ Open. 2026 Apr 20;16(4):e111824. doi: 10.1136/bmjopen-2025-111824 (PMC13110579; doi:10.1136/bmjopen-2025-111824)
Supplement: online supplemental file 3 [file bmjopen-16-4-s003.docx]

Ovid **Embase** <1974 to 2023 May 02>

Ovid **MEDLINE**(R) and Epub Ahead of Print, In-Process, In-Data-Review & Other Non-Indexed Citations, Daily and Versions <1946 to May 02, 2023>

1 exp clinical trial/ 2810241

2 randomi?ed control$ trial$.tw. 595987

3 randomi?ed controlled trial?.kw. 43936

4 clinical trial?.tw,kw. 1193058

5 controlled trial?.tw,kw. 717610

6 controlled clinical trial?.tw,kw. 76509

7 pragmatic trial?.tw,kw. 4768

8 complex intervention?.tw,kw. 8972

9 or/1-8 3926000

10 qualitative research/ 195657

11 qualitative research.tw,kw. 73428

12 (qualitative adj3 method$).tw. 96545

13 (qualitative method? or qualitative methodology).kw. 5908

14 (qualitative adj3 stud$).tw. 194458

15 qualitative study.kw. 7733

16 focus groups/ use ppezv 35576

17 focus group?.tw,kw. 141724

18 grounded theory/ 12883

19 grounded theory.tw,kw. 33487

20 narrative analys?s.tw,kw. 3969

21 process evaluation.tw,kw. 11433

22 mixed method?.tw,kw. 85963

23 mixed method$.mp. 87903

24 (in depth adj4 interview$).tw. 75352

25 in depth interview?.kw. 453

26 ((semi structured or semistructured) adj5 interview$).tw. 181156

27 semi structured interview?.kw. 709

28 mixed methodology.tw,kw. 1427

29 qualitative interview$.tw. 37648

30 qualitative interview?.kw. 1130

31 (interview$ and theme$).tw. 130624

32 interview?.kw. 6404

33 (interview$ and audio recorded).tw. 14748

34 qualitative case stud$.tw. 3810

35 descriptive case stud$.tw. 825

36 qualitative case study.kw. 75

37 descriptive case study.kw. 2

38 qualitative exploration.tw,kw. 4975

39 qualitative evaluation.tw,kw. 10865

40 qualitative intervention.tw,kw. 52

41 qualitative approach.tw,kw. 14587

42 qualitative inquiry.tw,kw. 2419

43 qualitativ$ analys$.tw. 60562

44 qualitative analysis.kw. 2513

45 (qualitative adj3 data).tw. 70081

46 qualitative data.kw. 237

47 discourse analysis.tw,kw. 5346

48 discursive.tw,kw. 5090

49 phenomenological.tw,kw. 48211

50 thematic analysis.tw,kw. 83982

51 ethnograph$.tw. 28263

52 ethnography.kw. 2778

53 action research.tw,kw. 11871

54 ethno?methodology.tw,kw. 219

55 social construction.tw,kw. 2280

56 or/10-55 774745

57 phenomenological characteristics.tw,kw. 390

58 phenomenological model.tw,kw. 2634

59 action research arm test.tw,kw. 1801

60 protocol.ti. 170304

61 or/57-60 175040

62 56 not 61 755277

63 9 and 62 49282

64 Patient Dropout/ use ppezv 8395

65 Patient Dropouts/ use oemezd 2033

66 Patient Recruitment/ use ppezv 69355

67 Research Subjects/ use oemezd 7568

68 patient recruitment.kw. 311

69 attrition.kw. 1902

70 patient retention.kw. 64

71 ((recruit$ or participat$ or take part or dropout$ or drop$ out$ or withdr?wl$ or barrier$ or retention or response$ or respond$ or attrition) adj10 trial?).tw. 212898

72 or/64-71 296566

73 63 and 72 7214

74 limit 73 to english language 7176

75 74 not abstract.pt. 5886

76 exp animals/ not human/ 10432543

77 exp nonhuman/ not humans/ 5822112

78 75 not (76 or 77) 5878

79 (202110* or 202111* or 202112*).dt. use ppezv 373760

80 (202110* or 202111* or 202112*).dc. use oemezd 474505

81 79 or 80 848265

82 78 and 81 163

83 limit 78 to yr="2022 -Current" 1007

84 82 or 83 1138

85 remove duplicates from 84 665

**Ovid APA PsycInfo** <2002 to April Week 4 2023>

1 clinical trials/ 11796

2 randomi?ed control$ trial$.tw. 43273

3 clinical trial?.tw. 37898

4 controlled trial?.tw. 48726

5 controlled clinical trial?.tw. 2966

6 pragmatic trial?.tw. 364

7 complex intervention?.tw. 1001

8 or/1-7 85578

9 qualitative research/ 10746

10 qualitative research.tw. 27709

11 (qualitative adj3 method$).tw. 31667

12 (qualitative adj3 stud$).tw. 76700

13 focus group?.tw. 41824

14 grounded theory/ 4422

15 grounded theory.tw. 17703

16 narrative analys?s.tw. 2869

17 process evaluation.tw. 1726

18 mixed method?.tw. 33572

19 mixed methodology.tw. 1005

20 (in depth adj4 interview$).tw. 30935

21 ((semi structured or semistructured) adj5 interview$).tw. 60044

22 qualitative interview$.tw. 12684

23 (interview$ and theme$).tw. 56679

24 interview?.tw. 265206

25 (interview$ and audio recorded).tw. 2605

26 qualitative case stud$.tw. 7101

27 descriptive case stud$.tw. 849

28 qualitative exploration.tw. 1758

29 qualitative evaluation.tw. 1106

30 qualitative intervention.tw. 16

31 qualitative approach.tw. 5358

32 qualitative inquiry.tw. 2333

33 qualitativ$ analys$.tw. 15359

34 (qualitative adj3 data).tw. 26382

35 discourse analysis/ 8573

36 discursive.tw. 10175

37 phenomenological.tw. 32724

38 thematic analysis.tw. 23317

39 ethnograph$.tw. 28514

40 action research.tw. 9180

41 ethno?methodology.tw. 485

42 social construction.tw. 3433

43 or/9-42 422135

44 phenomenological characteristics.tw. 190

45 phenomenological model.tw. 145

46 action research arm test.tw. 158

47 protocol.ti. 4311

48 or/44-47 4801

49 43 not 48 420849

50 experimental attrition/ 424

51 experimental recruitment/ 635

52 experimental subjects/ 3209

53 dropouts/ 521

54 ((recruit$ or participat$ or take part or dropout$ or drop$ out$ or withdr?wl$ or barrier$ or retention or response$ or respond$ or attrition) adj10 trial?).tw. 18623

55 or/50-54 22668

56 8 and 49 and 55 1116

57 (202110* or 202111* or 202112*).up. 44553

58 56 and 57 14

59 limit 56 to yr="2022 -Current" 98

60 58 or 59 110

**Cochrane Library Controlled Trial Register (CENTRAL)**

Search Name: QUART update

Date Run: 03/05/2023

ID Search Hits

#1 MeSH descriptor: [Qualitative Research] this term only 2051

#2 qualitative NEXT research:ti,ab,kw or qualitative NEXT method:ti,ab,kw or qualitative NEXT study:ti,ab,kw 5244

#3 MeSH descriptor: [Focus Groups] this term only 907

#4 MeSH descriptor: [Grounded Theory] this term only 41

#5 mixed NEXT method:ti,ab,kw or narrative NEXT analysis:ti,ab,kw 1043

#6 (interview):ti,ab,kw 27726

#7 qualitative case study:ti,ab,kw or descriptive case study:ti,ab,kw 5098

#8 qualitative NEXT exploration:ti,ab,kw or qualitative NEXT evaluation:ti,ab,kw or qualitative intervention:ti,ab,kw or qualitative approach:ti,ab,kw or qualitative analysis:ti,ab,kw 18816

#9 (qualitative data):ti,ab,kw 10369

#10 discourse analysis:ti,ab,kw or discursive:ti,ab,kw 197

#11 social construction:ti,ab,kw or action research:ti,ab,kw or ethnography:ti,ab,kw or thematic analysis:ti,ab,kw or phenomenological:ti,ab,kw 11053

#12 #1 or #2 or #3 or #4 or #5 or #6 or #7 or #8 or #9 or #10 or #11 53482

#13 MeSH descriptor: [Patient Dropouts] this term only 2214

#14 MeSH descriptor: [Patient Selection] this term only 5464

#15 ((recruit$ or participat$ or take part or dropout$ or drop$ out$ or withdr?wl$ or barrier$ or retention or response$ or respond$ or attrition) near/10 trial?):ti,ab,kw 70104

#16 #13 or #14 or #15 76794

#17 #12 and #16 4306

CENTRAL 3872

25/10/2021 on: 617

**EBSCO CINAHL**

S1 (MH "Clinical Trials+")

S2 TX randomized OR randomised OR trial*

S3 s1 OR s2

S4 (MH "Research Subjects+")

S5 TX ((recruit$ OR participat$ OR take part OR dropout$ OR drop$ out$ OR withdr?wl$ OR barrier$ OR retention OR response$ OR respond$ OR attrition) N10 trial?)

S6 s4 OR s5

S7 S3 AND s6

S8 (MH "Qualitative Studies+")

S9 (MH "Semi-Structured Interview") OR (MH "Structured Interview") OR (MH "Narratives")

S10 (MH "Focus Groups")

S11 TX qualitative N3 research OR TX qualitative N3 method* OR TX qualitative N3 study

S12 TX focus group* OR TX grounded theory OR TX narrative analysis

S13 TX mixed method* OR TX semi structured interview* OR TX in depth interview*

S14 TX qualitative exploration OR TX qualitative evaluation OR TX qualitative intervention* OR TX qualitative approach OR TX qualitative analysis OR TX qualitative data

S15 TX discourse analysis OR TX discursive OR TX thematic analysis OR TX ethnography OR TX action research OR TX phenomenological

S16 S8 OR S9 OR S10 OR S11 OR S12 OR S13 OR S14 OR S15

S17 s7 AND s16 **1512**

S18 s7 AND s16 Limiters - Published Date: 20171001-20221231 **196**

**Web of Science**: Social Science Citation Index

2: TS= ("thematic analysis") 32744

3: TS= ("social construction") 6707

4: TS= (discursive) 20179

5: TS= (discourse analysis) 44625

6: TS= (qualitative near/1 data) 27597

7: TS= (qualitative near/1 analysis) 40278

8: TS= (qualitative near/1 approach) 13835

9: TS= (qualitative near/1 intervention) 795

10: TS= (qualitative near/1 evaluation) 3029

11: TS= (qualitative near/1 exploration) 2422

12: TS= ("in depth interview*" or "semi structured interview*" or "qualitative interview*") 100878

13: TS= ("narrative analysis") 3099

14: TS= ("mixed method*") 43034

15: TS= ("grounded theory") 17342

16: TS= ("focus group*") 58166

17: TS= (qualitative NEAR/1 studies) 73321

18: TS= (qualitative NEAR/1 study) 73321

19: TS= (qualitative NEAR/1 method*) 34207

20: TS= (qualitative NEAR/1 research) 48735

21: #2 OR #3 OR #4 OR #5 OR #6 OR #7 OR #8 OR #9 OR #10 OR #11 OR #12 OR #13 OR #14 OR #15 OR #16 OR #17 OR #18 OR #19 OR #20 367171

22: TS=randomised controlled trial 114982

23: TS=randomized controlled trial 114982

24: #22 or #23 114982

25: TS=(("take part" or dropout$ or "drop$ out") NEAR/10 trial?) 218

26: TS=(( withdraw$ or barrier$ or retention or response$ or respond$ or attrition) NEAR/10 trial?) 6409

27: TS=(patient retention) 7682

28: TS=(patient attrition) 2004

29: TS=(patient dropout) 2566

30: TS=((recruit$ or participat$) NEAR/10 trial?) 1193

31: #25 OR #26 OR #27 OR #28 OR #29 OR #30 18672

32: #21 AND #24 AND #31 and Article or Review Article (Document Types) and 2023 or 2022 or 2021 (Publication Years) 76

**Proquest ASSIA**

Set# Searched for Results

S1 SU.EXACT("Cluster randomized trials") OR SU.EXACT("Clinical randomized controlled trials") OR SU.EXACT("Single blind randomized controlled trials") OR SU.EXACT("Cluster randomized controlled trials") OR SU.EXACT("Randomized controlled trials") OR SU.EXACT("Double blind randomized trials") OR SU.EXACT("Prospective controlled trials") OR SU.EXACT("Double blind randomized controlled trials") OR SU.EXACT("Clinical trials") 22772

S2 ti(randomized OR randomised) OR ab(randomized OR randomised) 38345

S3 s1 or s2 45567

S4 ti(qualitative OR ("focus group" OR "focus groups") OR interview* OR ("mixed method" OR "mixed methods") OR ethnography OR phenomenological OR "discourse analysis" OR discursive) OR ab(qualitative OR ("focus group" OR "focus groups") OR interview* OR ("mixed method" OR "mixed methods") OR ethnography OR phenomenological OR "discourse analysis" OR discursive) 159426

S5 s3 and s4 4553

S6 ti((recruit* N/10 trial?) OR (participat* N/10 trial?) OR ("take part" N/10 trial?) OR (dropout* N/10 trial?) OR (drop* out* N/10 trial?) OR (withdraw* N/10 trial?) OR (barrier* N/10 trial?) OR (retention N/10 trial?) OR (response* N/10 trial?) OR (respond* N/10 trial?) OR (attrition N/10 trial?)) OR ab((recruit* N/10 trial?) OR (participat* N/10 trial?) OR ("take part" N/10 trial?) OR (dropout* N/10 trial?) OR (drop* out* N/10 trial?) OR (withdraw* N/10 trial?) OR (barrier* N/10 trial?) OR (retention N/10 trial?) OR (response* N/10 trial?) OR (respond* N/10 trial?) OR (attrition N/10 trial?)) 5849

S7 s5 and s6 678

S8 (s5 and s6) AND pd(20211026-20231231) 66
