# Supplementary material for: Retention in trials: a qualitative evidence synthesis of studies reporting participant reasons for trial non-completion
Source: BMJ Open. 2026 Apr 20;16(4):e111824. doi: 10.1136/bmjopen-2025-111824 (PMC13110579; doi:10.1136/bmjopen-2025-111824)
Supplement: online supplemental file 4 [file bmjopen-16-4-s004.docx]

| **Table 1: Data extracted from each study** |
| --- |
| Author, Country, Date |
| *Host trial information:* Sample size, Unit of randomisation, Intervention, Comparator, Outcome, Follow-up period. |
| *Study information:* Aim, Methods, Who & Where conducted |
| *Study participants:* Number, Age, Gender, Ethnicity, Disease Type |
| Attrition behaviour: e.g. non-return of Q, non-return to clinic, from which trial group- Intervention or control? /Cross-over) |
| Definition of non-completion |
| Time points for interview  (e.g. retention period whether it looked at till end of host trial?) |
| Overview of study findings |
| Primary constructs (direct participant quotes) (from the results section of all studies that focussed on reporting related to data collection processes for trial outcome measures) |
| Secondary Constructs (authors interpretations) (from the results section of all studies that focussed on reporting related to data collection processes for trial outcome measures) |
