# Supplementary material for: Retention in trials: a qualitative evidence synthesis of studies reporting participant reasons for trial non-completion
Source: BMJ Open. 2026 Apr 20;16(4):e111824. doi: 10.1136/bmjopen-2025-111824 (PMC13110579; doi:10.1136/bmjopen-2025-111824)
Supplement: online supplemental file 5 [file bmjopen-16-4-s005.docx]

**Table 5. Characteristics of Included Studies.**

in some cases for the characteristics there was no clear distinction between the characteristics of participants who were deemed by the trial as non-retainers and participants who remained in the trial – indicted within the table

| **Paper** | **Aim** | **Gender** | **Age** | **Ethnicity** | **Country** | **Numbers of participants who data was from** | **Disease area** | **Attrition behaviour** | **Data collection methods** |
| --- | --- | --- | --- | --- | --- | --- | --- | --- | --- |
| **Nakash et al 2007 (1)** | To examine factors affecting response and non-response from the clinical trial participant’s perspective. | In this paper there is no specific characteristics for those who have withdrawn and – information from paper states the following –  “The sample consisted of 22 CAST participants, 11 male and 11 female individuals, aged from 16-62 years, with a mean age of 34 years. The majority of participants had reached two follow-up time points (4 and 12 weeks) and had therefore received two questionnaires. Fourteen participants had responded to all the questionnaires sent to them; eight participants had not responded to at least one of their follow-up questionnaires” | | Not clear | UK | We identified 7 participants were non retainers to at least one follow-up due to reasons not linked to the intervention  (8 non-retainers total) | Disease Area: Acute injury – severe ankle sprains  Intervention: Medical Device – different mechanical supports for severe ankle sprains | Non-response to postal questionnaire follow-up. 8 had not responded to at least one of their follow up questionnaires. | Semi structured interviews face to face (n=14) & telephone (n=8) |
| **Nicholas et al 2010 (2)** | To identify participants’ reasons for non-adherence to, and attrition from the online intervention | Of the 39 non-completers that were included.  22 were female.  17 males. | Of the 39 non-completers included.  20 were aged less than 30.  19 were aged 30 and over | Not clear | Australia | We identified 11 were non-retainers for reasons not linked to the intervention – unclear reporting –  (39 non-retainers total) | Disease Area: Mental Health - bipolar disorder  Intervention: Behavioural – online psycho-education programme either alone or with email support compared to a control group receiving online information. | Cessation of therapy sessions and/or non-completion of some or all intervention workbooks; non-completion of some or all control workbooks (online data collection) | Semi structured interviews - telephone |
| **Postel et al 2010 (3)** | To evaluate an e-therapy program with active therapeutic involvement for problem drinkers. Reasons for drop out were also investigated via a ‘dropout’ questionnaire consisting mainly of open questions. | In this paper there is no specific characteristics for those who have withdrawn and – information from paper states the following.  “Of the 156 participants who were included in the trial. Of these, 54% were women …..age ranged from 22-66 years with a mean of 45.3 years” | | Not clear | Netherlands | We identified 26 participants were non-retainers for reasons not linked to the intervention  (40 non-retainers total) | Disease Area: Problem drinkers  Intervention: Behavioural – e-therapy programme compared to waitlist control | Non-completion of treatment sessions and/or follow-up questionnaires. Non-retention was defined as anyone who did not complete the 3 month assessment. Dropouts in the e therapy group did not complete all 12 treatment sessions: 9 assignments and 3 assessments.  (online data collection) | A ‘dropout’ questionnaire consisting mainly of open questions |
| **Wells et al 2011 (4)** | To explore low income, minority cancer patient perspectives about not adhering or dropping out of depression treatment. | Dropouts were:  90% female | Dropouts were:  65% >50 years old | Dropouts were:  85% Latino | USA | 20 dropouts total – unclear reporting of the specific reasons as participants cited several reasons which did include reasons linked to the intervention | Disease Area: Mental Health – depression  Intervention: Mix – antidepressant medication and/or talk therapy compared to usual care | Cessation of problem-solving treatment sessions and/or antidepressant medication. PST dropouts were defined as patients who had fewer than 4 PST sessions. PST dropouts included those who initially agreed to be randomised to the intervention, but thereafter had either verbally declined treatment or did not show up for the therapy appointments. This included patients who had refused some sessions, but agreed to remain in the study for outcome interviews. Patients receiving antidepressant medication were dropouts if they discontinued treatment within 30 days (clinic visits) | In-depth telephone interviews |
| **Sanders et al 2012 (5)** | To explore barriers to participation and adoption of telehealth and tele-care from the perspective of people who declined to participate or withdrew from the trial | Male | 73 | Not clear | UK | We identified 1 participant was a non-retainer for reasons that did not link to the intervention  (3 withdrew total) | Disease area: Diabetes, chronic obstructive pulmonary disease, heart failure (trial included participants with various conditions but 1 participant whose quote we included had TID)  Intervention: Medical Device - General practices randomised to telehealth or telecare for their population’s vs waitlist control | Cessation of use of telehealth equipment or tele care devices (defined as withdrawing from the trial after joining the intervention arm).(online data collection) | Semi-structured interviews |
| **Henshall et al 2018 (6)** | To determine the overall experiences of newly diagnosed adults with T1D in an exercise study, and to understand issues that influence the retention of trial participants in such studies | Withdrawers.  2 males  2 females | Not specific to withdrawers but;  Aged ranged from 19-55 years | “All were of white British ethnic origin” | UK | We identified 3 participants were non-retainers for reasons not linked to the intervention  (4 participants withdrew total) | Disease Area: Diabetes (Type 1)  Intervention: Behavioural – exercise intervention vs usual care | No specific details given other than ‘those who withdrew before the end of the study’  (clinic visits) | Individual face to-face (n=6) and telephone interviews (n=14). |
| **Magazi et al**  **2014 (7)** | The VOICE-C sub study aimed to explore contextual barriers and facilitators to women’s use of the investigational product and  their experiences in the VOICE trial. | All females | Not clear but all participants aged between 18-40 years – with each quote you can see the age of the participant | Not clear | South Africa - Johannesburg | We identified 7 participants were non-retainers for reasons not linked with the intervention  (102 participants total) | Disease Area: HIV  Intervention type: Drug -Tenofovir 1% gel vs Tenofovir Disoproxil Fumarate tablet vs Emtricitabine/Tenofovir Disoproxil Fumarate tablet, placebo | The VOICE protocol defined a missed clinic  visit as non-attendance within the visit window (clinic visits) | Participants were randomly assigned to one of three interview modalities: a once-off in-depth interview (IDI);  serial ethnographic interviews (EI) repeated up to four times; or a focus group discussion (FGD) |
| **Newlands et al**  **2021 (8)** | To use the Theoretical Domains Framework (TDF) to identify barriers and enablers to participant retention in trials  requiring questionnaire return and/or attendance at follow-up clinics. | 9 were male  7 female | Aged ranged from 31-90 years (median=58) | All were White | UK | We identified 16 did not complete at least one follow-up due to reasons not linked to the intervention  (16 total) | Disease Area: Participants from 5 trials;   - Uncomplicated symptomatic Gallstone disease - Insomnia disorder - Urodynamic stress incontinence - Ureteric stones - Dental health   Intervention.  Surgical - Surgical – laparoscopic cholecystectomy vs conservative management  Extracorporeal shockwave lithotripsy as first treatment vs direct progression to ureteroscopy treatment  Device – male synthetic sling versus artificial urinary sphincter  Behavioural – online cognitive behavioural therapy trial  Complex; best dental recall interval | Participants had discontinued their trial participation for at  least one follow-up time point (i.e., they could have missed  any/all number of follow ups but had to have missed at  least one) either by not returning a questionnaire and/or  not attending a clinic visit within the preceding 12 months (clinic visits or postal questionnaires) | Semi structured telephone interviews |
| **Lawrie et al**  **2021 (9)** | to identify barriers and enablers to participant retention in trials requiring questionnaire return using the theoretical domains framework (TDF). | 2 male  7 female | 35-75 years old (median 53yrs) | Not clear | UK | 9 did not complete at least one follow-up due to reasons not linked to the intervention  (9 total) | Disease Area: Uncomplicated symptomatic Gallstone disease  Intervention: Surgical – laparoscopic cholecystectomy vs conservative management | Participants who had not returned at least one questionnaire at any time point during the study, and therefore had discontinued their follow-up at least once (postal questionnaires) | Semi-structured one-to-one interviews |
| **Kehagia et al 2022 (10)** | To outline a set of considerations informed a patient-centred approach to trial recruitment, retention and delivery | 5 males  3 females  Not clear who the 1 withdrawer is | 46-66 years | Not clear | UK | We identified 1 participant was a non-retainer for reasons not linked with the intervention  (8 withdrew total) | Disease Area: Parkinsons disease  Intervention: Drug – Simvastatin vs placebo | Included 8 participants “who commenced but subsequently withdrew from the trial”  (clinic visits) | Semi-structured interviews and focus groups with a subset of trial participants and their care partners. |
| **Draper et al 2025 (11)** | This study aimed to qualitatively examine retention in the Bukhali randomised controlled trial, from the perspective of trial participants and staff, through the lens of self-determination theory (SDT) | All women | Withdrawn participants were aged between 24-32 years old  Additional information about withdrawal  “Withdrawn participants – months enrolled in trial:  Mean ± SD = 16.53 ± 10.11 Median = 13.71  Range = 2.63 – 38.66” | Not reported | South Africa - Soweto | We identified 4 participants as non-retainers for reasons not linked to the intervention  (20 withdrew in total) | Maternal Health – Pregnancy related trial  Intervention;  The Bukhali **complex intervention** is delivered by trained community health workers, referred to as ‘health helpers’ (HHs). HHs provide health literacy support, conduct risk screening referral and management support, provide multimicronutrient supplementation and support health behaviour change through Healthy Conversation Skills | Withdrawal from the trial, unclear of specifics of the definition of withdrawal– reasons not linked to the intervention – as quotes all provided from withdrawers from the non-intervention group  Retention is defined by authors as follows;  Define retention: Staying in the trial, i.e. attending research visits, participating in intervention/control sessions. Being contactable is related to retention, because if they are not contactable, it is not possible to arrange visits, sessions etc.  Missing clinic visits | Focus Groups |

**References**

1. Nakash RA, Hutton JL, Lamb SE, Gates S, Fisher J. Response and non‐response to postal questionnaire follow‐up in a clinical trial–a qualitative study of the patient’s perspective. Journal of evaluation in clinical practice. 2008;14(2):226-35.

2. Nicholas J, Proudfoot J, Parker G, Gillis I, Burckhardt R, Manicavasagar V, et al. The ins and outs of an online bipolar education program: a study of program attrition. Journal of Medical Internet Research. 2010;12(5):e57.

3. Wells AA, Palinkas LA, Qiu X, Ell K. Cancer patients’ perspectives on discontinuing depression treatment: the “drop out” phenomenon. Patient preference and adherence. 2011:465-70.

4. Postel MG, de Haan HA, Ter Huurne ED, Becker ES, de Jong CA. Effectiveness of a web-based intervention for problem drinkers and reasons for dropout: randomized controlled trial. Journal of medical Internet research. 2010;12(4):e1642.

5. Sanders C, Rogers A, Bowen R, Bower P, Hirani S, Cartwright M, et al. Exploring barriers to participation and adoption of telehealth and telecare within the Whole System Demonstrator trial: a qualitative study. BMC health services research. 2012;12(1):1-12.

6. Henshall C, Narendran P, Andrews RC, Daley A, Stokes KA, Kennedy A, et al. Qualitative study of barriers to clinical trial retention in adults with recently diagnosed type 1 diabetes. BMJ open. 2018;8(7).

7. Magazi B, Stadler J, Delany-Moretlwe S, Montgomery E, Mathebula F, Hartmann M, et al. Influences on visit retention in clinical trials: insights from qualitative research during the VOICE trial in Johannesburg, South Africa. BMC women's health. 2014;14(1):1-8.

8. Lawrie L, Duncan EM, Dunsmore J, Newlands R, Gillies K. Using a behavioural approach to explore the factors that affect questionnaire return within a clinical trial: a qualitative study based on the theoretical domains framework. BMJ open. 2021;11(4):e048128.

9. Newlands R, Duncan E, Presseau J, Treweek S, Lawrie L, Bower P, et al. Why trials lose participants: a multitrial investigation of participants’ perspectives using the theoretical domains framework. Journal of clinical epidemiology. 2021;137:1-13.

10. Kehagia AA, North TK, Grose J, Jeffery AN, Cocking L, Chapman R, et al. Enhancing trial delivery in Parkinson’s disease: Qualitative insights from PD STAT. Journal of Parkinson's Disease. 2022;12(5):1591-604.

11. Draper CE, Tshetu N, Nkosi N, Lye S, Norris SA. Retention in the Bukhali trial in Soweto, South Africa: a qualitative analysis using self-determination theory. BMJ Global Health. 2025;10(2).
