# Supplementary material for: Retention in trials: a qualitative evidence synthesis of studies reporting participant reasons for trial non-completion
Source: BMJ Open. 2026 Apr 20;16(4):e111824. doi: 10.1136/bmjopen-2025-111824 (PMC13110579; doi:10.1136/bmjopen-2025-111824)
Supplement: online supplemental file 6 [file bmjopen-16-4-s006.docx]

**Supplementary File 6. Table of all first and second order constructs extracted**

| **Theme 1 Fluctuating Health** |
| --- |
| **1.1 Coming to terms with a diagnosis** |
| **First Order Constructs**  **Henshall et al.**  “Don’t think it kind of really sank in as to what I’d been diagnosed with … It had kind of hit me and I wasn’t really dealing with having it … I wasn’t taking my insulin and checking my levels as much … The doctors … Felt that it was best that I was taken off it. EXTOD 4 (female, Birmingham)”  **Nicholas et al.**  “I found it quite confronting, and reading the information made me feel uncomfortable, thinking that these issues related to me—I preferred the ostrich approach. [Male, 40-49 years, BEP group]”  “[I] found it difficult to sit down and do those things. I got into an anxiety and went off to do other things. I didn’t really want to sit down and think about it. [Female, 50-59 years, BEP+IS group]”  “I wasn’t ready to accept the illness. At that stage after diagnosis I wasn’t willing to change my life according to the program. [Male, 18-29 years, control group]” |
| **Second order Constructs**  **Henshall et al.**  “Some participants spoke of how they had struggled to come to terms with their T1D diagnosis, with one participant citing this as their reason for withdrawing from EXTOD. For this participant, her difficulties coping emotionally with her diagnosis had prompted her doctors to advise her to withdraw.”  **Nicholas et al.**  “Several participants reported that they found receiving weekly information about their disorder confronting or overwhelming. Many said they did not want to think about their illness and instead wanted to put it out of their minds”  “Some participants reported that they were not ready to accept their diagnosis of bipolar disorder and so didn’t relate to the program’s information and practical advice…..As the wider study was investigating the utility of the program in those newly diagnosed, some expressed the opinion that they may have enrolled in the program too soon after their diagnosis”  **Sanders et al.**  “Some respondents referred to the lack of integration of the trial with routine care and expressed surprise that doctors they consulted were not always aware of the trial (ID92)……..Another man (ID92) described the good care he received prior to joining the trial, but how he was subsequently discharged from the specialist professionals who had been involved in his care. Whilst he was entered into the trial for his diabetes, he described his main problems as ‘complex problems with my heart and breathing’, and that the faulty recordings and changes in service provision were causing him great stress”  **Wells et al.**  “Twelve of the 20 patients interviewed acknowledged that they had dropped out of treatment, citing several reasons and circumstances related to dropping out of treatment. These included the following……cancer-related (which included the emotional impact of the cancer diagnosis [n = 20]” |
| **1.2 Changeability to health** |
| **First Order Constructs**  **Kehagia et al.**  “I wasn’t prepared for how unpleasant ... and I think it’s a double whammy with Parkinson’s in that when the symptoms are pronounced, my dopamine level is low as well, not only am I physically uncomfortable, my brain and mood is less tolerant ... it’s just too much and I chickened out at that point (Withdrawn 6 54)”  **Lawrie et al.**  “Well, there were a few. I mean, I started off well and then because nothing had happened… I hadn’t had any pain or anything and then I’d put them aside and then somebody would send another one and say, ‘You haven’t completed it’. (Participant 2)”  **Nicholas et al.**  “The biggest problem I have with my bipolar disorder is consistency; when I’m down I can’t even brush my teeth or get up in the morning. So doing an education program with workbooks was beyond me. [Female, 18-29 years, BEP group]”  “A very short while after doing the program I fell into another episode, a depressive episode, and pretty much stopped doing everything, the program included. [Male, 18-29 years, BEP+IS group]”  “My highs interrupt my ability to see things through, and I get caught up in my highs. [Female, 30-39 years, Control group]”  “I often go walking when having highs because I have to keep moving, so I didn’t want to sit at a computer. [Male, 40-49 years, BEP+IS group]”  “I was so self-absorbed at the time that I was only interested in the information [rather than in returning workbooks]. [Male, 50-59 years, BEP+IS group]”  “Things really improved for me…I just felt really good and didn’t really feel like I had that much to offer in regard to finding out more about it. [Female, 30-39 years, control group]” |
| **Second Order Constructs**  **Kehagia et al.**  “Critically, a number of participants explained that they withdrew from the trial due to their adverse OFF experience and the prospect of future assessments.”  **Lawrie et al.**  “Most participants indicated that they did not perceive the questions within the questionnaire as relevant to their circumstances. This was because they were either not experiencing pain at the time of receiving the questionnaire or because they had been assigned to conservative management and therefore did not receive gallstone surgery and regarded the questions as irrelevant”.  **Nakash et al.**  “Almost half of the participants who did not respond to follow-up considered themselves to have made a full recovery by the second follow-up point.”  **Nicholas et al.**  “Many interviewees reported that, while they were able to complete the modules and workbooks when well, being in an acute phase of the illness interfered with their ability to participate in the program. Those in a depressive phase of the illness found the lack of energy and motivation common to depression a significant hurdle to completing the program”  “Participants who experienced episodes of mania during the study discussed how they became distracted by their manic symptoms and were unable to complete the online modules”  “Thus, the nature of the illness itself made it difficult for some participants to continue their involvement in the program. This was the most common theme in terms of reasons for discontinuation”  “Some participants reported ceasing to utilize the program after they gained what they wanted from it or once their mood had stabilized. Other participants indicated that they worked through the modules but did not complete the associated workbooks.”  “It was also reported by some participants that they no longer felt the need to participate in the program once their mood had stabilized and they were feeling well. This response was associated with another issue raised in the interviews, whereby a number of participants stated that they would reaccess the programs’ information after the study was completed if/when they were feeling depressed”  **Postel et al.**  “we could establish that in the e-therapy group…..6 [quit] because they were satisfied with the positive results being achieved (eg, “I have been sufficiently helped”)…..In the control group, 7 participants quit because they were satisfied with the results achieved” |

| **Theme 2 Balancing trial burdens** |
| --- |
| **2.1 Personal cost associated with trial participation** |
| **First order constructs**  **Draper et al.**  “I think time is the only thing. For an example I stay with my kids and they are still young, so for me coming here, I had to get someone to look after my kids, and sometimes you find that I tell a person that I am going to be gone for so long, but I end up taking much longer time here, which will then prevent the person to help me the next time when I ask for help”. (Withdrawn participant—non- intervention)”  **Henshall et al.**  “I left … Because I didn’t have enough time … The study is of such a long duration and I just found that too challenging with work … If you had appointments at eight o’clock in the evening every time you needed me then so be it … I have to do a hell of a lot of juggling in order to fit that sort of stuff in. EXTOD 17 (male, Bristol)”  **Magazi et al.**  “Yes, it is the issue of time. Sometimes you would find that as a person you have other plans, and they get spoilt because you have to come here to the study and find [do] their long visits. When you have to attend your visits then all your plans have to stop, and that is really irritating, hey. I once missed my visit and I ended up telling myself that I won’tbe part of the study anymore because I missed my visit (age 20-25, EI).”  **Newlands et al.**  “Yeah, childcare was a big problem for me. I had to go out to the clinic, so driving within a city environment, so getting parked and then getting to the actual clinic itself. There was a bit of a walk from the carpark. (Female, mid 40’s, recruited through Twitter)”  “I think maybe the travelling to different hospitals, and different bits and pieces, shall we say, complicated it a little bit … When I went to Bristol, yes, I live 170-odd miles away. … (Male, mid 50’s, MASTER study)”  “It (clinic visit) wouldn’t have been a priority, hence why I didn’t go in the time slots they asked me, because I wasn’t prepared to prioritise it over other appointments during work time. (Female, 30-35yrs, INTERVAL study)” |
| **Second order constructs**  **Draper et al.**  “Limited social support  The absence of social support was mentioned by some participants, which negatively influenced their ability to stay involved in the trial, and are therefore relevant to retention. This was to do with not having someone to take care of their other child/children when attending the research site (or not being able to pay someone to look after their child), expectations of family members to receive some or all of the financial incentive that participants receive (mentioned above) or family members not being supportive of the health behavioural changes encouraged by the trial HHs. Conversely, participants reported receiving social support from trial staff, which helped make up for the lack of support at home.”  **Henshall et al.**  “These time pressures had led to two of the interviewed participants withdrawing from EXTOD prior to completion”  **Magazi et al.**  “More than half (57%) of VOICEC participants were employed as servers in restaurants and hotels, cashiers and packers in supermarkets, security guards, cleaners, administrators, and receptionists. In many of these situations, the hours were long and presented difficulties for attending regular monthly clinic visits. Even those who worked night shifts were too tired or had other domestic responsibilities to attend to during the day.”  “An unmarried participant found a job as a nurse, and missed five consecutive clinic visits because of her demanding working hours and infrequent time off (age 21-25, FGD).”  **Nakash et al.**  “work commitments which participants felt were the cause of their non-response  **Wells et al.**  “Twelve of the 20 patients interviewed acknowledged that they had dropped out of treatment, citing several reasons and circumstances related to dropping out of treatment. These included the following………financial issues [n = 11], …. employment related concerns [n = 11], and caregiving demands [n = 3]),” |
| **2.2 Type and timing of data collection** |
| **First order constructs**  **Henshall et al.**  “I’m not bothered for taking blood or doing injections or anything like that … But I can remember feeling bothered by that at the time. I think because I was very ill, really, still, and still quite fragile … I can remember that upsetting me … Because it was a study and because I didn’t have to do it … If you volunteered to have a blood test and it takes a few times for them to take the blood, it’s kind of like putting yourself through something difficult that you didn’t really need to do. EXTOD 6 (female, Leeds)”  **Lawrie et al.**  “No, I suppose you have to be able to read, you have to be able to understand, take the time and comprehend what it’s asking you as well. And also, to recollect, to recall when you’ve gone to the doctors, all that sort of thing, you know? (Participant 7)”  “I suppose if the questions had been really complicated or difficult to answer, maybe. Or if you just can’t remember what exactly has happened over the time. I suppose the time between questionnaires, if it’s every three months then that’s not too bad, but if it was longer than that then I’d definitely have trouble remembering exactly what symptoms I’d had and how many times and all the fine details. (Participant 1)”  “The questionnaire thing for me is a barrier because I’ll think, ‘Oh, I’ll put it to the side, I’ll do it when I’ve got time,’ and I never get the time. I’ve got my second one to fill and it’s been there for months. Really bad! (Participant 2)”  “I much preferred it when I could give oral answers to a simplified version in which she said, “These are just the main questions that we need to know.” That didn’t take nearly as long, and it was quite focused and straightforward. (Participant 2)”  “It [the questionnaire] wasn’t very interesting, it was pretty boring (Participant 3)”  **Newlands et al.**  “Two, but I think they were repeated. Same one. (Male, early 50’s, CGALL Study)”  “No, because I recognised that it was from the study, so it wasn’t a surprise. But at the same time I wasn’t expecting it, does that make sense? … I think, it’s pretty bad, I’m guessing three (questionnaires)… That’s a complete guess … I wouldn’t say confidently. It could be four… that’s really bad, isn’t it? I don’t know! I don’t know how many forms I’ve filled out. They’re all a bit the same, it’s very repetitive (Female, late 30’s, CGALL study)”  “I thought it was a bit monotonous, know what I mean? Yeah, it was easy to fill out and easy to follow but it wasn’t very… I don’t know what I was expecting, to be honest. (Female, late 30’s, CGALL study)”  “I think the fact it was online, and it was very clear. The instructions were very clear, the questions were very clear. They weren’t overly lengthy questionnaires either. But, no, the whole thing was very good. (Female, age unknown, DISCO study)”    “I could have filled it in online, but personally given a choice between filling it in online and being able to have the form in front of me to fill in, I was quite happy with the form. (Male, early 70’s, INTERVAL Study)”  “That (online follow-up) wasn’t a problem because it meant I could reply in the evening time or when I wasn’t at work (Female, age unknown, DISCO Study)  “I didn’t think that it would, but it did. It did work for me because it allowed me to do it as and when I wanted to, and you could complete small sections of it at a time” (Female, late 50’s, DISCO Study)  “It was a sit down and do it, sort of thing. It wasn’t the most engaging but then I don’t know that questionnaires can be, to be honest….No, I did it, but maybe not as quickly as I should have, do you know what I mean? It got left on the table for a little bit” (Female, late 30’s, CGALL Study)  “I haven’t read it through, I just looked at it. It could be bigger print….I think doing the questionnaire over the phone may have helped” (Male, 50-55yrs, CGALL Study)  “if they did that (call to complete over the phone), if they asked me the questions I would have filled the questionnaire in that way” (Male, 50-55yrs, CGALL Study)  “I think I’m quite old fashioned, I do quite like pen and paper, the physical thing” (Female, late 30’s, CGALL Study) |
| **Second order constructs**  **Lawrie et al.**  “Participants also reported confusion over receiving the same questionnaire on multiple occasions. As they did not expect to receive (or understand the purpose of) identical questionnaires at different time points, they did not send some of the questionnaires back to the trial office”  “The majority of participants recognised that there were certain skills required to complete the questionnaires, such a remembering the level of pain experienced over a certain amount of time.”  “Some also acknowledged that completing the questionnaire required concentration, and sometimes this was reported as a barrier to completion and return.”  “Preference for questionnaire format differed among participants: some regarded postal administration as convenient for them, others indicated that they would have preferred to speak to someone or complete the questionnaire electronically”  “Some participants linked their preference for completing the questionnaire with a trial member of staff to their social personality. The majority of participants perceived the questionnaire to be boring, and most indicated that if it were a lengthy questionnaire, they would struggle to complete it.”  **Nakash et al.**  “A second participant felt that the questionnaire was too long and time consuming and this discouraged her from responding.”  “External reasons were beyond the inﬂuence of the trial and included such things postal strikes.” |
| **2.3 Strategies to support continued participation** |
| **First order constructs**  **Draper et al.**  “It was nice, really nice, starting with the transport that you guys offer for us, it made us feel special being picked up from home”. (Withdrawn participant—non- intervention)”  **Lawrie et al.**  “I would actually think, “Well, I’ve got to get this done” and do it and then send it off. Saying that, yes, probably… You’re right, I would probably say, “Oh, I’m going to sit down this evening, fill this questionnaire in and then get it sent off’.” So, yes, in that way, there was a plan. (Participant 5)”  “Yes, like I say, once I received it, I filled it in and on the way to pick my son up from school I put it in a post box. (Participant 8)”  “Yeah, I just want to do it because I want to do it. I don’t really want anybody to try and bribe me to do it or encourage me to do it due to any kind of incentive, no. In some ways, actually, that would put me off. (Participant 3)”  “It’s an extra thing that we volunteered to do, I could have just said no to my surgery anyway and not gone for this, do you understand what I mean, because I had that choice. But I went for this and I volunteered for it, just that little bit of a reminder and a bit of support, “We’re helping you out on this, it’s okay, I know it’s late but can you try and get it in now. Or do you want to come and see me?” There’s a few more options. That’s it. (Participant 6).”  “I think once I didn’t return it and they sent me out a reminder questionnaire, which was good. It wasn’t a telling off, but it was just a reminder that it was there. That was good if you know what I mean…Yeah, without being forceful, kind of thing. (Participant 4)”  “Once I’ve filled it in, my husband supports me in terms of he gets it to you, as in he posts it for me because I don’t go out to post it when I’ve got work or whatever. But that’s the only support…my husband is understanding. He understands the pain and he helps me when I’m suffering and stuff, but no, I don’t have any other support, not really. (Participant 6)”  “No, I’ve got no pressures from anyone. Nobody encourages me either. At first, my sister half encouraged me to take part because I was about 90% sure I was going to do it, and then after talking to her and a couple of others, just things that were said I just said, “Yeah, I’ll do it”. (Participant 1)”  **Magazi et al.**  “He fetched me from my house…he asked me why didn’t I go because they [study staff] said I missed my visit. I said my phone is not working. So he brought me here [to the trial clinic] (age 18-21yrs, FGD)”  **Newlands et al.**  “I was quite happy because it’s a lot nearer to me than the LH (previous dental surgery) one was. (Male, late 60’s, INTERVAL study)  “The questionnaires were really easy, and they came with a stamped envelope, was easy to post back, so there was no inconvenience for me. I completed it, it was really quick, really easy to read, really well laid out and, yeah, they sent an envelope for me to send it back so that was no problem at all. Yeah, I was happy to do it. (Female, mid 30’s, INTERVAL study)”  “Yeah, they did invite me for a clinic appointment, and they gave me certain slots, times slots, but due to work commitments I wasn’t able to make those slots. So, I gave them a call and asked if we could rearrange different slots and they weren’t able to do that, so I wasn’t able to complete the study. (Female, mid 30’s, INTERVAL study)”  “Different ones I had to do, I could see from the schedule what I was going to be doing that day, but just sometimes I was waiting a long time, and then I’d be late for getting my kids from school. … I’d have to wait for one doctor, and then the nurse would say, “I’ve taken your medication out of the fridge, and it would have to be out of the fridge for a while before I inject you”, just things like, things weren’t lined up. (Female, mid 40’s, recruited through Twitter)”  “Actually, the trouble has been that my appointments have been put off quite a lot because of the change of -- they’re expanding. They’ve been building another, adding another room to the practice and it has led to appointments being shuffled about quite a lot.. (Female, 85-90yrs, INTERVAL study)”  “Maybe a free check-up at the dentist would have been nice. (Female, mid 30’s, INTERVAL study)”  “No, no. It (incentives or rewards) would have put me off. (Female, 85-90yrs, INTERVAL study)”  “I would just put the date in my calendar and that would be bit it (Female, mid 30’s, INTERVAL Study)”  “I don’t know, maybe a text message if you’ve got a hospital appointment, to say, “Are you going to confirm for this appointment, or not?” ( Male, mid 50’s, MASTER study)”  “If I’ve got to attend a clinic, I would put it in my notes on my phone, or calendar, so it notifies me. (Male, mid 50’s, MASTER Study)”    “… it’s quite a long journey now, but I always combine it with doing other things … It’s just like a day out … (Male, early 70’s, INTERVAL study)  “It just felt like I had no support, like a buddy system or something like that would be nice. (Female, mid 40’s, recruited through Twitter)”  “Yes, I put them on my kitchen calendar. (Female, 85-90yrs, INTERVAL Study)”    “I leave that to my husband. There’s nothing to make it (returning a questionnaire) easier, but at the same time it wasn’t difficult either. … I’m not one for paperwork, so … it’s not something I look forward to. (Male, early 50’s, CGALL study)”    “The questionnaires were really easy, and they came with a stamped envelope, was easy to post back, so there was no inconvenience for me. I completed it, it was really quick, really easy to read, really well laid out and, yeah, they sent an envelope for me to send it back so that was no problem at all. Yeah, I was happy to do it. (Female, mid 30’s, INTERVAL study)”  “I think once I didn’t return it and they sent me out a reminder questionnaire, which was good. It wasn’t a telling off, but it was just a reminder that it was there. That was good, if you know what I mean.… Yeah, without being forceful, kind of thing. (Female, late 30’s, CGALL study)”  “I did ask my husband if he’d help me do the questionnaire, but he said, “Yes, I’ll do it, yes, I’ll do it” (Female, mid 30’s, INTERVAL Study)”    “No (reward or incentives would have required). I think if you’re going to do these things then do them willingly. (Female, late 50’s, DISCO study)  “I don’t want to be bribed to do it. (Female, 85-90yrs, INTERVAL Study)”  “The other thing that was really good was that, I think, occasionally they sent us some vouchers. I wasn’t expecting that. That was very much appreciated. … it’s like a little voucher to say thank you for participating, and that was good. …I think the vouchers were good. I think the other thing would be, that would be encouraging as I mentioned before, is when the survey is completed just to have a little note with a few bullet points on about what the findings from the survey were (Male, early 70’s, INTERVAL study)”  “They sent me £15 worth of vouchers, Love To Shop vouchers, on just one occasion. I wasn’t expecting them because I didn’t finish the study. (Female, 30-35yrs, INTERVAL Study)”  “I always had it out in a visible place, so it was always on my coffee table. A visual reminder (Female, late 30’s, CGALL study)”  “No, not really (used any strategies). I’m pretty good at responding to emails, I like to just get stuff done rather than have it hanging around. So, there was no strategy as such. (Female, age unavailable, DISCO Study)”  “Reminder post it notes, no. I just try to remember. (Male, early 50’s, CGALL Study)”  “No, I haven’t forgot to return any questionnaires. … No, there isn’t anything that makes it harder in that I always make sure that I’ve got it in my diary, and I allocate the time. (Male, early 70’s, INTERVAL study)” |
| **Second order constructs**  **Lawrie et al.**  “Some participants indicated strategies that they had used to help them complete the questionnaires. These included ‘internal’ strategies such as making a plan to complete and return the questionnaires and keeping a note of symptoms to ensure accurate reporting.”  “Some participants indicated that they completed the questionnaires whenever they had spare time and would often complete the questionnaire as soon as they received it to avoid forgetting about it”  “Participants also mentioned external strategies to encourage questionnaire return. The majority of participants reported that they did not expect to receive any incentives or rewards for completing and returning the questionnaires, and for one participant rewards/incentives offered would have been perceived as a form of bribery”  “Most participants suggested that they would have liked to receive a prompt or reminder to complete and return the questionnaires……However, one participant indicated that they had received a friendly reminder from the trial office and believed it to be effective because it was not communicated in a forceful manner.”  “A couple of participants reported that they received support from family members to return the questionnaires, while the majority indicated that they completed and returned the forms independently. All participants indicated that they received some level of support from the trial office to complete trial- related activities. This included receiving gentle reminders or facilitating questionnaire completion over the phone with a trial staff member.”  “Participants suggested that they did not feel any pressure to complete and return the questionnaires, and for some this was a barrier to questionnaire completion and return”  **Magazi et al.**  “For instance when the outreach team could not contact a participant on her phone after she missed her visit they contacted her partner (she had given consent for the clinic to contact her partner) and he promised to bring her to the clinic”  **Nakash et al.**  “All non-responders who expressed an opinion about incentives felt that an incentive would probably have encouraged them to respond.”  **Wells et al.**  “Twelve of the 20 patients interviewed acknowledged that they had dropped out of treatment, citing several reasons and circumstances related to dropping out of treatment. These included the following…… systems-related (which included service-related logistical issues [n = 13]” |

| **Theme 3 Navigating life as a trial participant** |
| --- |
| **3.1 Life gets in the way** |
| **First order constructs**  **Newlands et al.**  “Well, time is a pressure today for everybody, isn’t it? We all lead such busy lives. (Male, early 70’s, MASTER Study)”  “I mean there were other life-changing events going on around that time, so it was more things that were happening personally, I think, that life just got busier. (Female, late 50’s, DISCO Study)”  “Of course that’s (partner being unwell) taken on a couple of roles, I’m the driver now and of course I’m looking after him. Looking after things that he’s always looked after before, I don’t think it’s really interfered me in taking part in the research programme. (Female, mid 70’s, CGALL Study)”  “Of course, I didn’t fill it out, I was moving house, I was living in temporary accommodation, I had a lot of problems with this house I’ve just built and, quite frankly, it was right down the list. I couldn’t be bothered with it, quite frankly. (Male, early 70’s, MASTER Study)”  **Nicholas et al.**  “I didn’t have the time, and with everything else, it wasn’t a priority.” [Female, 18-29 years, control group] |
| **Second order constructs**  **Draper et al**  “some of the withdrawn participants spoke about other commitments that make it difficult to stay, such as employment (or focus on seeking employment), studies…”  **Magazi et al.**  “ A college student described college life as ‘hectic’; she missed two visits and ran out of tablets (age 18-21yrs, IDI)”  **Nakash et al.**  “These were reasons [for non-retention] such as pregnancy, exams..”  **Nicholas et al.**  “Time-related factors such as being too busy or life being too hectic were also given as reasons for discontinuation. Such reasons included being busy at work, moving house after signing up for the program, and having more important focuses.”  **Postel et al.**  “we could establish that in the e-therapy group 11 participants dropped out because of personal reasons unrelated to the e-therapy program or the study (eg, ill family member).. In the control group….2 [participants quit] for personal reasons.  **Wells et al.**  “Twelve of the 20 patients interviewed acknowledged that they had dropped out of treatment, citing several reasons and circumstances related to dropping out of treatment. These included the following……cancer treatment commitments [n = 11]),” |
| **3.2 Perceptions of self** |
| **First order constructs**  **Nakash et al.**  “‘forgetful’, ‘disorganised’ and ‘lazy’”  “. . . is there a particular reason why you found it difﬁcult to do them for us or . . .  Do you know what . . . laziness I’m just gonna put it down to that Ok and em it wasn’t because you were disgruntled about part of the project  Deﬁnitely not no Ok. No deﬁnitely not. (1103 SG p5)  **Newlands et al.**  “I’m slightly anxious, but not overly anxious. Just in case anything needs to be done (while visiting a dentist) …” (Male, early 30’s, INTERVAL Study)  “To be honest I’m no good with paperwork.”  “It’s kind of like, “What’s this person going to think of me? Am I answering it right?” (Female, late 50’s, DISCO Study)”  **Nicholas et al.**  “I have issues with procrastination. I suppose laziness is the only reason. [Female, 18-29 years, control group]” |
| **Second order constructs**  **Nicholas et al.**  “However, lack of motivation was also a commonly mentioned reason, such as being forgetful or lazy about completing the program” |
| **3.3 Cultural community context** |
| **First order constructs**  **Draper et al.**  “Because I felt like when I speak to my family members, like they were judging me or wanted to tell me how to go about doing things, even if I wanted to do the things the way I wanted to, so that I can make my own mistakes, learn from my mistakes. (Withdrawn participant—non- intervention)” |
| **Second order constructs**  **Magazi**  “A 35-yearold woman missed four visits to attend her husband’s funeral in Zimbabwe and to observe the mourning period and cleansing rituals. Another younger participant said that her father ‘forced her’ to visit home regularly, resulting in frequent missed VOICE visits (age 18-21yrs, EI).”  **Wells et al.**  “Twelve of the 20 patients interviewed acknowledged that they had dropped out of treatment, citing several reasons and circumstances related to dropping out of treatment. These included the following……cultural (which included language communication problems [n = 7]…. discrimination from providers [n = 5])” |

| **Theme 4 Managing expectations of participation** |
| --- |
| **4.1 Expectations of care and relational support offered by trial participation** |
| **First order constructs**  **Draper et al.**  “The lady that recruited me had said that she would call me, but she didn’t call me. She called me after some months after I had not been here and she wanted to know if I was no longer interested in the study, I told her that I was interested but I wasn’t getting any communication from her, and she said that she had been calling me but was not getting through, so maybe I had not charged the phone”. (Withdrawn participant—non- intervention)”  **Lawrie et al.**  “I’m not sure if it’s just myself or if other people are on the same, medical management (conservative management), a little bit more information, a little bit more contact with someone asking, ‘How are you doing? Have you received this information?’ or just giving information out would help a bit more. I’m not sure if it’s… if you don’t do it, I didn’t know that medical management was just filling in questionnaires. I thought there’d be a little bit more to it, that’s all. (Participant 8)”  **Magazi et al.**  “Hmm, what I like about the VOICE staff members is that they are more patient than any other studies that I have ever participated in. Like, at the beginning of the year I was busy at work and I missed my clinic visit. I thought maybe they have withdrawn me from the study. I was so surprised to receive a call from the VOICE staff member who said ‘you can come tomorrow and after the clinic visit we will give you a lift to work. The nurses will also give you the clinic attendance letter.’ The VOICE staff members encouraged me to continue with the study because before they phoned me I thought that I have messed up everything and I do not qualify to be in the study anymore. They are very supportive (age 25-30, EI)”  **Newlands et al.**  “There was no care involved, it was just two questionnaires. (Female, early 30’s, INTERVAL Study)”  “I really felt as if there was no acknowledgement or any appreciation for my taking part…(Female, mid 40’s, recruited through Twitter)”  “Yeah, I felt a bit anxious about if the nurse was going to be mean to me again. I didn’t look forward to going to the visits” (Female, mid 40’s, recruited through Twitter)  “Yeah, I just felt like I wasn’t engaged in it. … I thought I was doing a good thing, and it didn’t feel like a good thing at all. Yeah, it became- going once a month. (Female, mid 40’s, recruited through Twitter)”  “I don’t think there was any benefits of me doing it really because I didn’t get -- because the questions I asked I didn’t get a response. (Female, mid 30’s, INTERVAL Study)”  “So I tried to add that to it and I suggested someone phone me or whatever, but I didn’t get any response, so I thought, “Well, mmm”, I lost a bit of interest… (Male, mid 70’s, MASTER Study)”  “when I returned the questionnaire it would’ve been nice to receive a small note saying, “Thank you Mr X, we’ve received the questionnaire and it’s going to be included as part of the study”, or just a little recognition that the document had been received and it was now going to be processed as part of the study. (Male, mid 70’s, MASTER Study)”  “I wrote a letter, a little note alongside it and popped that in and said I’d welcome a telephone call or a conversation about the study at that point, and I received no reply. So to be truthful I lost a bit of interest at that point. (Male, mid 70’s, MASTER study)” |
| **Second order constructs**  **Draper et al.**  “Some participants spoke about not hearing from the trial staff, and given the challenges mentioned above, it is possible that staff’s attempts to reach them were not successful. This was more of an issue for participants who had withdrawn—most not necessarily due to no longer wanting to participate in the trial.”  **Lawrie et al.**  “Importantly, participants suggested that the activities involved in the trial did not meet their initial expectations, with some indicating that they expected a greater level of medical attention, such as support and information provided to help manage their condition, such as dietary advice, among participants randomised to conservative management.”  **Wells et al.**  “Twelve of the 20 patients interviewed acknowledged that they had dropped out of treatment, citing several reasons and circumstances related to dropping out of treatment. These included the following……patient-provider problems [n = 10])” |
| **4.2 Understanding what is expected as a trial participant** |
| **First order constructs**  **Lawrie et al.**  “I think at the appointment initially, they said that the trial had been extended. They didn’t give me a timeframe for how long it would go on for. (Participant 1)”  “It’s been playing on my mind that I haven’t done it yet. Not willing me but making me angry with myself for not doing it…For messing up the trials. (Participant 3)”  “Well, you’ve completed a job, you know that it’s important, it helps in clinical trials, it’s helping finding out stuff. That’s the upside of doing it, and you feel satisfaction, that you’re making a difference. (Participant 4)”  **Newlands et al.**  “No, I didn’t think I would make much difference at all, to be honest. I was very doubtful about the value of it (attending a clinic appointment). … Well, it’s just a small contribution that would probably add up to something at the end of the day, but it was a very small contribution. (Female, 85-90y, INTERVAL study)”  “Well the dental information, if they didn’t get information from patients they won’t be able to improve you know. (Male, late 60’s, INTERVAL study)”  “What would have happened? Well, nothing happened. I still got the voucher. Yeah, it’s just a shame for me, if I hadn’t have started the trial then I wouldn’t have minded but because I started it and then wasn’t able to finish it (missed clinic appointments), it’s a bit disappointing” (Female, mid 30’s, INTERVAL study)  “If I came upon a question that I wasn’t happy with, I wouldn’t answer it. I’m quite free to do that. It was explained very well I think, that you are under no obligation and that I could pull out from it all at any time. Knowing that, I was quite happy with things. (Female, mid 70’s, CGALL Study)”  “No, clearly I didn’t because I only filled in the first one so I probably made no difference at all to the MASTER study. (Male, mid 70’s, MASTER study)”  “Nothing, I suppose. I didn’t think anything would happen. (Female, 85-90y, INTERVAL Study)”  “I honestly don’t know whether I have made a difference to the study. (Male, mid 50’s, MASTER Study)”  “That’s the upside of doing it, and you feel satisfaction, that you’re making a difference. (Female, late 30’s, CGALL study)”  It was pretty high because I thought if it helps the study into the problem, it can help others. (Female, early 50’s, DISCO Study)”  I mean the fact that I was incontinent was very important to me, and the fact that something might be done about it was very important to me, … (Male, mid 70’s, MASTER Study)”  “Not willing me but making me angry with myself for not doing it (returning the questionnaire). … For messing up the trials. … If they don’t do it, they might upset the trials, and just make a loss of money doing the trial. (Male, early 50’s, CGALL Study)”  **Wells et al.**  “For example, one participant said, “Did I dropout? No, I didn’t dropout. I became busy and I figured I started missing calls.” Similarly, another said, “Like I have repeated, I didn’t stop any treatment.”  “Another participant expressed that she had not chosen to dropout so therefore was not comfortable with this label, “It wasn’t dropping out because I didn’t not want to get help … It’s not like it was under my power. You know what I mean? You can say [dropout] if you want to but I don’t feel like I want[ed] to get out of the study.”  “Similarly, another participant thought the label sounded negative, “I think that I dropped out but I don’t know – it does not sound good.” Some participants did not understand what was being asked and called the question “weird;” one participant said that her continuation with other treatment meant that she had not dropped out. One participant said, “I don’t know what to tell you. I’m confused. I can’t answer that question. You can ask me a thousand times. I can’t answer it.” |
| **Second order constructs**  **Lawrie et al.**  “The majority of participants reported being unaware of the duration of their participation within the trial, although some indicated a certain level of awareness of their ongoing participation”  “Participants reported feelings of guilt triggered by questionnaire non- return. This was mostly linked to the belief that they were letting the trial team down and having a negative effect on trial results.”  “Around half of the participants reported a sense of satisfaction brought about by successfully completing and returning the questionnaires and felt it was their duty to complete this task as a trial participant. Feelings of satisfaction were mostly linked to their beliefs about consequences: that their contribution would help the research”  **Nakash et al.**  “The majority of participants who failed to respond were keen to stress that their lack of response was through no fault of the trial itself and they were happy to continue to be involved. This suggests a lack of understanding of the importance of complying with follow-up to the outcome of the trial…..Of the eight participants who had failed to respond to the questionnaires, four had either a poor or questionable understanding of the trial”  **Wells et al.**  “The remaining eight patients either disagreed that they  had dropped out of treatment, perceived the term to be misleading because it implied a willful or voluntary response to an uncontrollable situation, or were confused by the term and uncertain as to whether it applied to their own situation.” |
