# Supplementary material for: Retention in trials: a qualitative evidence synthesis of studies reporting participant reasons for trial non-completion
Source: BMJ Open. 2026 Apr 20;16(4):e111824. doi: 10.1136/bmjopen-2025-111824 (PMC13110579; doi:10.1136/bmjopen-2025-111824)
Supplement: online supplemental file 7 [file bmjopen-16-4-s007.docx]

**Supplementary File 7. CASP Quality assessment**

| **Study ID** | Was there a clear statement of the aims of the research? | Is a qualitative methodology appropriate? | Was the research design appropriate to address the aims of the research? | Was the recruitment strategy appropriate to the aims of the research? | Was the data collected in a way that addressed the research issue? | Have the relationship between the research and participants been adequately considered? | Have ethical issues been taken into consideration? | Was the data analysis sufficiently rigorous? | Is there a clear statement of findings? | How valuable is the research? | Overall assessment |
| --- | --- | --- | --- | --- | --- | --- | --- | --- | --- | --- | --- |
| Nakash et al (1) | Yes | Yes | Yes | Yes | Yes | Not reported | Yes | Yes | Yes | Useful findings – NB: Limited 1st order construct data perhaps because paper also included data from trial retainers (and this seemed to be main emphasis)  *We identified 7 participants who were non retainers to at least one follow-up due to reasons not linked with the intervention – unclear reporting of how many of the 7 were reported.*  *(8 non-retainers total)* | Minor concerns |
| Nicholas et al (2) | Yes | Yes | Yes | Yes | Yes | Not reported | Yes | Yes | Yes | Useful - richer in data compared to other studies, helps to build understanding of reasons for non-retention.  *We identified 11 were non-retainers for reasons not linked to the intervention – unclear reporting –*  *(39 non retainers total)* | Minor concerns |
| Wells et al (3) | Yes | Yes | Yes | Yes | Yes | Not reported | Not reported | Unclear – very brief details and presented in quantitative manner | Yes | Less useful Qualitative data presented was very limited both 1st and 2nd order constructs) and was discussed in a more quantitative way.  *20 dropouts total – unclear reporting of the specific reasons as participants cited several reasons which did include reasons linked to the intervention.* | Minor concerns |
| Postel et al (4) | Yes | This reported some qualitative data from open ended questionnaire response options | Yes | Yes | Not reported for the qualitative data | Not reported | Yes | Unclear– not mentioned other than that the data was systematically analysed – reflects that is was not a qualitative study as such | Yes | Less useful Qualitative data presented was very limited (both 1st and 2^nd^ order constructs) and was discussed in a more quantitative way.  *We identified 26 participants were non-retainers for reasons not linked to the intervention* | Minor concerns |
| Sanders et al (5) | Yes | Yes | Yes | Yes | Yes | Not reported | Not reported | Yes | Yes | Less useful Very thin data provided and very few 1st order constructs  *We identified 1 participant was a non-retainer for reasons did not link the intervention*  *(3 withdrew total)* | Minor concerns |
| Henshall et al (6) | Yes | Yes | Yes | Yes | Yes | Yes | Unclear – informed consent mentioned but not ethical approvals | Yes | Yes | Useful findings – NB: Limited 1 st order construct data. *We identified 3 participants were non-retainers for reasons not linked to the intervention*  *(4 participants withdrew total)* | Minor concerns |
| Magazi et al (7) | Yes | Yes | Yes | Unclear – briefly outlined in the paper but more details provided in a separate publication | Yes - but uncertainty as to why different methods of interviews and focus groups were used | Not reported | Unclear – ethics has been provided but due to the setting of the research in an LMIC setting more information on context specific ethical issues may be needed | Yes but briefly described | Yes | Useful; provides insights into retention issues in low-middle income settings. NB  Limited first order constructs reported.  *We identified 7 participants were non-retainers for reasons not linked with the intervention*  *(102 participants total)* | Minor concerns |
| Lawrie et al (8) | Yes | Yes | Yes | Yes | Yes | Unclear - limited reporting | Yes | Yes | Yes | Useful;  Researchers outline the importance of the findings, it adds to existing evidence etc and clearly identifies new areas for development and how it can be transferred for other trial settings.  NB; 9 did not complete at least one follow-up due to reasons not linked to the intervention | Minor concerns |
| Newlands et al (9) | Yes | Yes | Yes | Yes | Yes | Not reported | Yes | Yes | Yes | Useful findings; Researchers outline the importance of the findings, it adds to existing evidence etc and clearly identifies new areas for development and how it can be transferred for other trial settings  Lots of rich data and first order constructs provided  NB; We identified 16 did not complete at least one follow-up due to reasons not linked to the intervention | Minor concerns |
| Kehagia et al (10) | Yes | Yes | Yes | Yes | Yes | Not reported | Yes | Yes | Yes | Less useful - very limited first order constructs provided. NB; results included data from participants but also care partners and researchers  *We identified 1 participant was a non-retainer for reasons not linked with the intervention*  *(8 withdrew total)* | Minor concerns |
| Draper et al (11) | Yes | Yes | Yes | Yes | Yes | Yes | Unclear- ethics has been provided but due to the setting of the research in an LMIC setting more information on context specific ethical issues may be needed | Yes | Yes | Useful Findings within the low-middle income setting to help understand retention issues in this context, NB; thin data provided on non-retainers for reasons not linked to the intervention. NB: 20 participants withdrew from the trial and participated in the focus group  However the number that we could clearly identify as withdrawing for reasons not linked to the intervention include 4. | Minor concerns |

References

1. Nakash RA, Hutton JL, Lamb SE, Gates S, Fisher J. Response and non‐response to postal questionnaire follow‐up in a clinical trial–a qualitative study of the patient’s perspective. Journal of evaluation in clinical practice. 2008;14(2):226-35.

2. Nicholas J, Proudfoot J, Parker G, Gillis I, Burckhardt R, Manicavasagar V, et al. The ins and outs of an online bipolar education program: a study of program attrition. Journal of Medical Internet Research. 2010;12(5):e57.

3. Wells AA, Palinkas LA, Qiu X, Ell K. Cancer patients’ perspectives on discontinuing depression treatment: the “drop out” phenomenon. Patient preference and adherence. 2011:465-70.

4. Postel MG, de Haan HA, Ter Huurne ED, Becker ES, de Jong CA. Effectiveness of a web-based intervention for problem drinkers and reasons for dropout: randomized controlled trial. Journal of medical Internet research. 2010;12(4):e1642.

5. Sanders C, Rogers A, Bowen R, Bower P, Hirani S, Cartwright M, et al. Exploring barriers to participation and adoption of telehealth and telecare within the Whole System Demonstrator trial: a qualitative study. BMC health services research. 2012;12(1):1-12.

6. Henshall C, Narendran P, Andrews RC, Daley A, Stokes KA, Kennedy A, et al. Qualitative study of barriers to clinical trial retention in adults with recently diagnosed type 1 diabetes. BMJ open. 2018;8(7).

7. Magazi B, Stadler J, Delany-Moretlwe S, Montgomery E, Mathebula F, Hartmann M, et al. Influences on visit retention in clinical trials: insights from qualitative research during the VOICE trial in Johannesburg, South Africa. BMC women's health. 2014;14(1):1-8.

8. Lawrie L, Duncan EM, Dunsmore J, Newlands R, Gillies K. Using a behavioural approach to explore the factors that affect questionnaire return within a clinical trial: a qualitative study based on the theoretical domains framework. BMJ open. 2021;11(4):e048128.

9. Newlands R, Duncan E, Presseau J, Treweek S, Lawrie L, Bower P, et al. Why trials lose participants: a multitrial investigation of participants’ perspectives using the theoretical domains framework. Journal of clinical epidemiology. 2021;137:1-13.

10. Kehagia AA, North TK, Grose J, Jeffery AN, Cocking L, Chapman R, et al. Enhancing trial delivery in Parkinson’s disease: Qualitative insights from PD STAT. Journal of Parkinson's Disease. 2022;12(5):1591-604.

11. Draper CE, Tshetu N, Nkosi N, Lye S, Norris SA. Retention in the Bukhali trial in Soweto, South Africa: a qualitative analysis using self-determination theory. BMJ Global Health. 2025;10(2).
