# Supplementary material for: Retention in trials: a qualitative evidence synthesis of studies reporting participant reasons for trial non-completion
Source: BMJ Open. 2026 Apr 20;16(4):e111824. doi: 10.1136/bmjopen-2025-111824 (PMC13110579; doi:10.1136/bmjopen-2025-111824)
Supplement: Supplementary file 8 [file bmjopen-2025-111824.reviewer_comments.pdf]

PEER REVIEW HISTORY

BMJ Open publishes all reviews undertaken for accepted manuscripts. Reviewers are asked to complete a checklist review form and are provided with free text boxes to elaborate on their assessment. These free text comments are reproduced below.

ARTICLE DETAILS

Title (Provisional)

Retention in trials: A Qualitative Evidence Synthesis of studies reporting participant reasons for trial non-completion

Authors

Murphy, Ellen; Gillies, Katie; Skea, Zoe; Biesty, Linda; Hunter , Andrew; Noor, Nurulamin M; McCann, Sharon

---

VERSION 1 - REVIEW

Reviewer 1

Name Searle, Aidan

Affiliation National Institute for Health and Care Research, Bristol Biomedical Research Centre

Date 13-Nov-2025

COI None

This manuscript reports conducting a qualitative evidence synthesis (QES) of studies reporting participant reasons for trial non-completion. In the main it is a valuable, well structured and coherent manuscript and is based on following the well established QES methodology of Thomas and Harden.

That said, there are a number of points that if addressed will further enhance the quality of the paper and assist potential readers understanding of the process and the issues raised in the manuscript.

Firstly, with regard to the strengths and limitations bullet points feels a bit contradictory when referring to the 'thinness of the data' not supporting study findings as the study findings are thin as they based on the 'moderate' quality of the contributing studies.

With regard to the methods the authors refer to 'primary quotes' and 'author interpretations', however in the field / wider literature the established terms are 'first order' and 'second order constructs' respectively. By referring to and defining these terms it will serve to distinguish from reference to 'primary data' throughout the manuscript.

In relation to this it would be good to see a table in appendices that shows all the data extraction and labelled as first order /second order constructs.

The data extraction form is provided but it is not clear if this form was designed specifically for this QES or was adapted from an existing form elsewhere? Please clarify.

It is great to see such meaningful analytical themes as developing such themes is a key challenge in QES. However, it is not clear how these themes were arrived at and would benefit from further explanation of the process.

I also wondered if the qualitative studies presented in the original papers selected for the review were part of process evaluations or more focused on the participant experiences of RCTs?

With regard to further Discussion I wondered if the findings were or could be considered from the perspectives / implications of the design of the RCT (i.e double blind, crossover etc) and /or the type of intervention in the treatment arm. For example, a drug trial might be considered to be a more passive experience from the participant perspective whereas a behavioural intervention might require a higher level of active engagement and potentially more interaction with trialists / intervention providers. It would be good to see some discussion of these points in relation to pre-trial explanations / information giving etc.

I also feel that this QES would have benefitted from some PPI perhaps in the development of analytical themes and the implications of the findings and how the issues relating to trial non-completion described in the paper might be addressed?

Finally, are there any ethical issues that arise from the findings relating to trial non-completion that should be raised in the discussion to inform the recruitment and retention in RCTs?

**Reviewer**	**2**
**Name**	**Menezes Junior, Antonio**
**Affiliation**	**UFG, Internal Medicine**
**Date**	**08-Feb-2026**
**COI**	**None**

Important comments

1. Clear understanding of important terms

The paper could benefit from clearer definitions of "non-retention," "trial non-completion," and "non-completion of outcome data collection" at the beginning. Although differences become more apparent later, clearly describing these concepts in the introduction would enhance conceptual clarity and accessibility for a wide readership.

2. Transferability and applicability

The synthesis encompasses 11 studies and 105 non-retained participants from diverse settings. While this is recognized, the discourse might be enhanced by elaborating on:

o Generalizability to extensive multicenter RCTs

o Differences in healthcare systems and settings

o How it relates to modern trials that use remote or digital follow-up

A brief discussion of how this affects modern trial design could make the manuscript more useful in practice.

3. Reflexivity and openness in synthesis

Please think about going into more detail about reflexivity. A brief statement delineating the study team's expertise in trial methodology and the strategies employed to mitigate interpretive bias would enhance methodological transparency, in accordance with contemporary qualitative synthesis reporting standards.

4. Understanding what CERQual found

The CERQual evaluations are suitable. The conversation could be clearer about what "moderate confidence" implies for trialists and which results are most useful for planning interventions to keep people in the study.

Minor comments

• Clarify whether a review protocol was registered (e.g., PROSPERO or OSF).

• Provide a brief justification for excluding non-English studies after an unrestricted search.

• Consider simplifying and enlarging text in Figure 2 to improve readability in print.

• The abstract could include the number of included studies and participants to improve completeness.

• Minor language edits would improve flow, although the manuscript is already written in clear and acceptable English.

VERSION 1 - AUTHOR RESPONSE

**Reviewer Comment**	**Authors' Response**
**Reviewer 1:**	
This manuscript reports conducting a qualitative evidence synthesis (QES) of studies reporting participant reasons for trial non-completion. In the main it is a valuable, well structured and coherent manuscript and	Thank you for this encouraging feedback and for the constructive suggestions, which we have addressed as requested, in the specific

is based on following the well established QES methodology of Thomas and Harden. That said, there are a number of points that if addressed will further enhance the quality of the paper and assist potential readers understanding of the process and the issues raised in the manuscript.	point by point responses outlined in the sections below.
Firstly, with regard to the strengths and limitations bullet points feels a bit contradictory when referring to the 'thinness of the data' not supporting study findings as the study findings are thin as they based on the 'moderate' quality of the contributing studies.	Thank you for this useful feedback. We have completely revised the Strengths and Limitations (after the abstract) to focus on the methods.<br><br>**Added new text**<br><br>Strengths and limitations of this study (after the abstract section; (pgs 3-4)<br><br>• **To our knowledge, this is the first synthesis of qualitative studies reporting on participant reasons for trial non-completion, linked to outcome data collection.**<br>• **This synthesis was conducted using established, standardised, and reproducible qualitative evidence synthesis methods.**<br>• **We used the GRADE-CERQual approach to provide a transparent assessment of our confidence in the review findings.**<br>• **The synthesis included 11 eligible studies, from 14 separate trials, predominantly in high-income settings which may limit the transferability of findings.**
With regard to the methods the authors refer to 'primary quotes' and 'author interpretations', however in the field / wider literature the established terms are 'first order' and 'second order constructs' respectively. By referring to and defining these terms it will serve to distinguish from	Thank you for this feedback. We have now edited our QES methodological sources as requested to reflect 'first order constructs' (to refer to participants' own words, accounts as reported in the original included qualitative studies) and 'second order constructs' (to refer to the interpretations

reference to 'primary data' throughout the manuscript.<br><br>In relation to this it would be good to see a table in appendices that shows all the data extraction and labelled as first order /second order constructs	provided by the original study authors based on participants accounts).<br><br>**New added text**<br><br>Methods<br><br>Additionally, for all quotes presented we indicate whether the quote was a primary participant quote **(first order constructs)** or the study author's interpretation reported in the primary study **(second order constructs)** (pg 22).<br><br>Results<br><br>Replacement of 'primary quote' to '**first order construct'** across Results (pages 24-38).<br><br>Replacement of 'authors interpretation' to **'second order construct'** across Results (pages 24-38).<br><br>We have now added a new table in the appendices that shows all data extraction labelled as first and second order constructs.<br><br>**New added text**<br><br>**A table showing all the data extracted as first and second order constructs, is presented in Supplementary File 6.** (pg 11)
The data extraction form is provided but it is not clear if this form was designed specifically for this QES or was adapted from an existing form elsewhere? Please clarify.	Two review authors (SM and EM) extracted data from all included studies using a pre-designed data extraction form, **adapted and expanded from the Skea et al QES [10], to focus on participants' reasons for trial non-completion specifically related to outcome data collection.** (pg 10)
It is great to see such meaningful analytical themes as developing such themes is a key challenge in QES. However, it is not clear how these themes were arrived at and would benefit from further explanation of the	Thank you for positive feedback. We have expanded our explanation of the analytical theme development process.<br><br>**New added text**

process.	Methods: Data synthesis (pg 12)<br><br>Codes were grouped into overarching themes with sub-themes, to support analytical development with team members. **A draft summary of findings across the included studies was undertaken by SM and EM and shared with all review authors to facilitate collective interpretation. Up to this stage, the themes reflected descriptive accounts of the original findings.** Following team discussions, SM and EM **then** developed more analytical themes to **move beyond** the original data, consistent with the approach outlined by Thomas and Harden [18]. Through **ongoing team discussions, constant comparison of NVivo codes, and refinement of conceptual groupings, broader analytical themes were generated to encapsulate all the descriptive themes.** (pg 12)
I also wondered if the qualitative studies presented in the original papers selected for the review were part of process evaluations or more focused on the participant experiences of RCTs?	The qualitative studies presented in the original papers selected, focused on participant experiences of RCTs.
With regard to further Discussion, I wondered if the findings were or could be considered from the perspectives / implications of the design of the RCT (i.e double blind, crossover etc) and /or the type of intervention in the treatment arm. For example, a drug trial might be considered to be a more passive experience from the participant perspective whereas a behavioural intervention might require a higher level of active engagement and potentially more interaction with trialists / intervention providers. It would be good to see some discussion of these points in relation to pre-trial explanations / information giving etc	Thank you for this helpful comment. We have now added text to address the points raised.<br><br>**New added text**<br><br>Discussion (pg 38)<br><br>**Although certain aspects of trial design may influence how participants perceive the relevance of engaging with outcome data collection, the evidence presents a mixed picture, with influences operating at multiple levels. Factors such as clinical specialty, the acute or chronic nature of the condition under study, intervention complexity, multimorbidity, and the relational dynamics between participants and site staff may each shape, to varying**

	**degrees, participants' engagement with completing outcome measures.**<br>**Although trial interventions were not the focus of this review, the type of intervention could also potentially influence how participants engage with completing outcome data collection**<br><br><br>(pg 39) **As such, potentially differing expectations of engagement with data collection, reinforce the need for clear, tailored pre-trial explanations about what data will be collected, why it is important, and the expected time and effort involved. Ensuring participants have a realistic understanding of these requirements may not only support more informed decision making, but also support more complete outcome data collection.**
I also feel that this QES would have benefitted from some PPI perhaps in the development of analytical themes and the implications of the findings and how the issues relating to trial non-completion described in the paper might be addressed?	We acknowledge the value of including patient and public involvement (PPI) in this QES. However, we were unable to undertake PPI activity for this review due to the absence of dedicated funding or resources to support meaningful involvement. As such, we have added this as a limitation of the review.<br><br>**New added text**<br>Strengths and limitations<br><br>**Finally, we were unable to undertake PPI activity for this review due to the absence of dedicated funding or resources to support meaningful involvement. We acknowledge this as a limitation and would plan, in future updates of the review, to secure the necessary resources to enable PPI input.** (pg 43)
Finally, are there any ethical issues that arise from the findings relating to trial non-completion that should be raised in the	Thank you for highlighting this as an important consideration for the Discussion section. We have now added details about

discussion to inform the recruitment and retention in RCTs?	ethical implications arising from the findings in relation to non-completion to help inform interventions to support retention in RCTs.<br><br>**New added text**<br><br>Discussion (pg 40-41)<br><br>**From an ethical perspective, issues related to trial non-completion raise important questions about how to balance participants' right to withdraw voluntarily with the responsibility to ensure participation decisions are well-informed and not perceived as potentially coercive. While it is essential that trial participation remains voluntary, providing clear information about what trial involvement entails, including expectations about outcome data collection, would support more informed decision making and lessen ethical tensions.**<br><br>**These issues highlight the need for future research to explore how best to communicate expectations without exerting undue influence, ensuring that decisions remain both fully informed and voluntary.**
**REVIEWER 2**	
**Important comments**	
1. Clear understanding of important terms. The paper could benefit from clearer **definitions of "non-retention," "trial non-completion," and "non-completion of outcome data collection"** at the beginning. Although differences become more apparent later, clearly describing these concepts in the introduction would enhance conceptual clarity and accessibility for a wide readership.	Thank you for this helpful suggestion. We have now added earlier descriptions in the manuscript as suggested, to improve clarity for the reader.<br><br>**New added text**<br><br>Background<br><br>Retention **(i.e., ensuring that trial participants remain in a trial to provide primary outcome data)** in randomised controlled trials (RCTs), herein referred to as

	trials, is an ongoing challenge for trial teams. (Pg 5)<br><br>Trials **commonly experience problems with missing data often referred to as a 'loss to follow-up', 'drop out' or 'attrition' [2, 3]. This means that trials often experience non-completion of outcome data collection (i.e. fail to obtain complete primary outcome data)** from participants, a problem that has important potential implications for utility of results from these trials. (pg 5).<br><br>A meta-ethnography by Skea et al [10], was the first to synthesise qualitative evidence on participant-**reported factors influencing non-retention in trials, focusing** on intervention adherence in addition to trial processes**.** (pg 6)<br><br>*Rationale for this Qualitative Evidence Synthesis (pg 6)*<br><br>The Qualitative Evidence Synthesis (QES) reported in this paper advances the discussions generated by the Skea et al [10] meta-ethnography, with a focus on trial non-completion linked to trial processes **(i.e. data collection for outcome measures).**
2. Transferability and applicability.<br>The synthesis encompasses 11 studies and 105 non-retained participants from diverse settings. While this is recognized, the discourse might be enhanced by elaborating on:<br>o Generalizability to extensive multicenter RCTs<br>o Differences in healthcare systems and settings<br>o How it relates to modern trials that use remote or digital follow-up<br>A brief discussion of how this affects modern trial design could make the manuscript more useful in practice.	We appreciate your thoughtful suggestions We have now incorporated some additional text in the Discussion to address the points raised.<br><br>**New added text**<br><br>*Implications for research*<br><br>**It is widely recognised that variations such as, healthcare settings, trial types, clinical conditions, may shape retention in different ways.** (Pg 44)<br><br>*Discussion* (Pg 41)<br><br>**Advances in digital technology may create additional opportunities to support**

	**participants with follow-up data collection, and the implications of these developments for trial design are important to consider. However, any proposed solutions require careful exploration at the design stage to ensure approaches remain methodologically robust, ethically sound, and acceptable to participants and do not introduce unintended consequences e.g. remote method of data collection could negatively impact on retention if participants are motivated by seeing the clinician in a hospital for follow up**.<br><br>**The studies included in this review were primarily conducted in the UK, however, there was representation of trial participants from low-middle income settings in South Africia [25, 28] and the USA [27]. Including perspectives from ethnic minority and lower socioeconomic groups is important for understanding trial non-retention issues and developing culturally sensitive strategies to support trial retention, linked to supporting outcome data collection.**
3. Reflexivity and openness in synthesis<br>Please think about going into more detail about reflexivity. A brief statement delineating the study team's expertise in trial methodology and the strategies employed to mitigate interpretive bias would enhance methodological transparency, in accordance with contemporary qualitative synthesis reporting standards	We have now added a more detailed reflexivity statement to enhance methodological transparency.<br><br>**New text added**<br><br>**Reflexive note** (pg 19)<br><br>**The review team included researchers with a range of experiences in trial design, conduct and retention research, which will have shaped the review processes. To help mitigate interpretive bias, we held ongoing team discussions, reflected on how our backgrounds might shape the synthesis, and considered alternative explanations when comparing concepts across studies.**

	**Throughout the review we also documented the synthesis stages, team discussions, and rationale for analytic and methodological decisions to enhance transparency and ensure that the resulting interpretations remained grounded in the data.Core review activities were undertaken by four authors (EM, SM, KG and LB) with all authors contributing to ongoing interpretation and synthesis through regular team meetings and shared online resources.**
4. Understanding what CERQual found<br>The CERQual evaluations are suitable. The conversation could be clearer about what "moderate confidence" implies for trialists and which results are most useful for planning interventions to keep people in the study.	Thank you for this helpful point — we have now added clearer explanation of the GRADE-CERQual assessments<br>and what the "moderate confidence" implies for trialists.<br><br>**New text added**<br><br>Discussion (pg 41-42)<br><br>**Using the GRADE-CERQual Assessment of Confidence in the Evidence, the 'moderate confidence' across findings indicates that the review team considers that it is likely that the review finding is a reasonable representation of the phenomenon of interest, albeit with some concerns, particularly regarding the adequacy of the data relating to the richness and quantity presented in some studies. Using the GRADE-CERQual approach to provide a transparent assessment of confidence in the review findings, may help trialists identify more easily which areas to prioritise for potential intervention development to support trial retention.**
Minor comments	

Clarify whether a review protocol was registered ((e.g., PROSPERO or OSF).	No review protocol was registered for this QES as it was part of a review update.
Provide a brief justification for excluding non-English studies after an unrestricted search.	We had initially considered that it might be necessary to limit the inclusion of non-English studies if the search yielded a large number of them. However, this did not occur, and so no studies were excluded on the basis of language after the unrestricted search. We have removed this sentence about non-English studies being excluded. (pg 9).
Consider simplifying and enlarging text in Figure 2 to improve readability in print.	We have now improved readability of Figure 2 as requested.
The abstract could include the number of included studies and participants to improve completeness.	Thank you – we have now added to the abstract the number of included studies, participant numbers and dates of when the studies were undertaken.<br><br>**New added text**<br><br>Abstract<br><br>**Participants: We identified 11** studies **reporting qualitative data from** 14 separate trials, **with findings from** 105 trial non-retainers. **The studies were undertaken between 2007 and 2025**. (pg 2)
Minor language edits would improve flow, although the manuscript is already written in clear and acceptable English.	We have made a number of language edits throughout the manuscript to improve flow and clarity. These are all shown via track changes in the marked manuscript.

VERSION 2 - REVIEW

Reviewer 1

Name Searle, Aidan

Affiliation National Institute for Health and Care Research, Bristol Biomedical Research Centre

Date **19-Mar-2026**

COI

I am satisfied that the authors have revised the manuscript in response to all the reviewers comments and is now acceptable for publication in BMJ Open.
